# Vav2 is a master regulator of repair against bacterial pore-forming toxins

**DOI:** 10.26508/lsa.202603633

**Published:** 2026-07-09

**Authors:** Victor Gbenga Kayejo, Ashlee Hensley, Tejal Katore, Peter A Keyel

**Affiliations:** Department of Biological Sciences, Texas Tech University , Lubbock, TX, USA

## Abstract

Despite antibiotic therapy, 25–35% of patients with necrotizing soft tissue infections (NSTIs) die. The etiologic agents for NSTIs include *Streptococcus pyogenes* and *Clostridium perfringens*, which secrete the cholesterol-dependent cytolysins (CDCs) streptolysin O and perfringolysin O to disrupt cell membranes. Whereas cells resist this damage by activating Ca^2+^-dependent repair pathways, including mitogen-activated protein kinase kinase (MEK)–dependent microvesicle shedding, dysferlin-mediated patch repair, and annexin-mediated membrane clogging, the upstream regulators of these responses have remained elusive. Here, we demonstrated that the Rac guanine nucleotide exchange factor Vav2 accounts for almost all of Ca^2+^-dependent repair against CDCs. Inhibiting or knocking down Vav2 sensitized multiple cell types to CDCs, whereas blocking other Rac guanine nucleotide exchange factors did not. Mechanistically, Vav2 triggered the critical mixed lineage kinase 3-MEK–dependent repair pathway. MEK activation rescued repair in Vav2-inhibited cells. Blocking dysferlin or annexins failed to increase damage beyond Vav2 inhibition, suggesting Vav2 coordinates multiple repair pathways. Thus, Vav2 controls multiple Ca^2+^-activated repair pathways that protect cells from CDCs produced during NSTIs.

## Abstract (precis)

This one protein accounts for almost all Ca2+-dependent repair by activating at least 3 distinct pathways.

## Graphical Abstract

**Figure:**
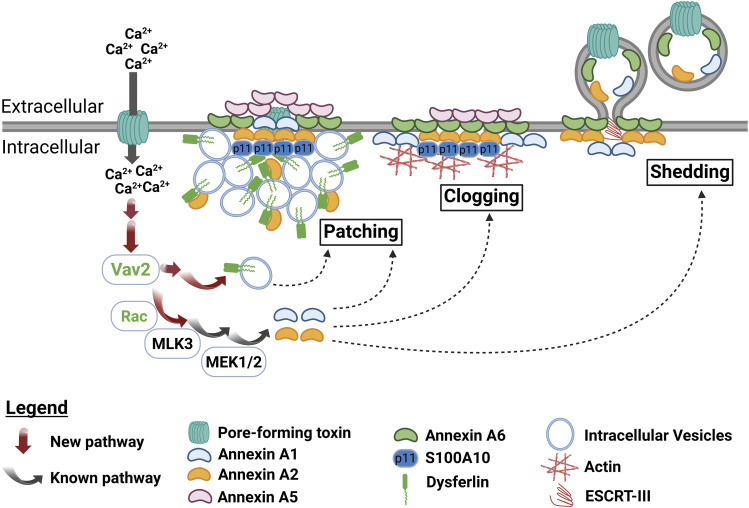

## Introduction

Severe bacterial infections that destroy skin, muscle, and soft tissue kill 20–40% of patients, even with medical care (Hakkarainen et al, 2014; Jabbour et al, 2016). These necrotizing soft tissue infections are caused by bacteria such as *Streptococcus pyogenes* and *Clostridium perfringens* (Thapa et al, 2020). The virulence of these bacteria comes in part from the secreted pore-forming toxins (PFTs) streptolysin O (SLO) and perfringolysin O (PFO) (Limbago et al, 2000; Awad et al, 2001). SLO and PFO are cholesterol-dependent cytolysins (CDCs), so they bind to cholesterol and create pores in the cell membrane, thereby causing membrane damage (Thapa et al, 2020). Damage to epithelial, endothelial, and immune cells enhances bacterial spread into tissues and evasion of the immune response (Los et al, 2013; Thapa et al, 2020). Failure to resolve membrane damage leads to cell death, so it is crucial to understand how cells repair membrane damage.

Membrane repair describes a collection of potentially interrelated biological processes that reseal the membrane. Resealing mechanisms can vary by cell type, source of damage, and extent of damage, which complicates pathway determinations (Cooper & McNeil, 2015; Jimenez & Perez, 2017). In general, cells sense membrane damage by ion flux, especially Ca^2+^ (Cooper & McNeil, 2015; Jimenez & Perez, 2017). Upon damage, three Ca^2+^-activated repair mechanisms reseal the membrane by clogging the breach, patching the breach, and/or sequestering and eliminating the breach by shedding damaged membranes as microvesicles (Thapa et al, 2020). Clogging is mediated by annexins and transglutaminases, which form crystalline lattices and plugs to block the breach (Roostalu & Strahle, 2012; Demonbreun et al, 2016b; Jimenez & Perez, 2017). Patch repair is the hetero- and homotypic fusion of vesicles with the membrane, driven by C2 domain–containing proteins, including synaptotagmins, copines, and the muscle-specific protein dysferlin (Chakrabarti et al, 2003; McNeil et al, 2005; Alves et al, 2022). Microvesicle shedding is the sequestration and shedding of PFTs on microvesicles, controlled by lipids (Keyel et al, 2011), endosomal sorting complex required for transport recruitment (Jimenez et al, 2014), and mitogen-activated protein kinase kinase (MEK) activation (Ray et al, 2022). Elevation of ceramide and other proteins can protect cells from damage (Schoenauer et al, 2019; Ray et al, 2022; Haram et al, 2023) via unknown mechanisms. Although previously thought to be a repair mechanism, we and others showed that endocytosis occurs after repair to clean up the damage (Keyel et al, 2011; Romero et al, 2017; Nygard Skalman et al, 2018; Schoenauer et al, 2019; Thapa & Keyel, 2023; Stefl et al, 2024). The relative weights of these repair pathways vary by toxin type because patch repair is more important to resist the small pore-forming toxin aerolysin, whereas microvesicle shedding is more critical to stop CDCs (Thapa & Keyel, 2023). The signaling pathways coordinating these pathways downstream of Ca^2+^ influx are unknown.

Because many repair proteins contain Ca^2+^-binding domains, one hypothesis is that Ca^2+^ influx is sufficient to drive repair. For example, dysferlin, annexins, and copines all contain Ca^2+^-binding domains and are recruited to damage sites (Demonbreun et al, 2016a; Boye et al, 2017; Alves et al, 2022; Kayejo et al, 2023). However, live cell imaging shows that cells are flooded with Ca^2+^ throughout the cell upon damage (Babiychuk et al, 2009, 2011; Thapa & Keyel, 2023). With elevated Ca^2+^ throughout the cell, it is unclear how Ca^2+^-sensitive proteins are targeted specifically to the site of damage. Therefore, additional signaling pathways are needed to coordinate the repair response.

Our recent work showed that an atypical MAP kinase signaling pathway involving mixed lineage kinase 3 (MLK3) and MEK, but not extracellular regulated kinase (ERK), accounts for 70% of Ca^2+^-dependent repair. This signaling axis triggers annexin A2 membrane translocation and microvesicle shedding (Ray et al, 2022). When this pathway is blocked, annexin A1 and annexin A6 try to compensate by translocating to the membrane faster (Ray et al, 2022). However, the upstream signaling proteins that activate MLK3 remain unknown.

One potential upstream signaling partner active during membrane repair is the Rho GTPase family. Rho GTPases such as Rho, Rac, and cdc42 are each implicated as signaling proteins that promote actin remodeling and wound healing after damage in *Xenopus* oocytes (Benink & Bement, 2005; Moe et al, 2021) and mammalian cells (Iliev et al, 2007; Lam et al, 2018). These small GTPases have many downstream interaction partners, positioning them to execute cell signaling programs in response to extracellular stimuli like damage. Notably, both cdc42 and Rac interact with MLK3 via the latter’s cdc42/Rac interactive binding motif (CRIB motif) (Du et al, 2005). Activation of these GTPases is further controlled by guanine nucleotide exchange factors (GEFs). For example, Rac1 can be activated by Vav proteins, T-lymphoma invasion and metastasis-inducing protein-1 (Tiam1), Trio, DOCK1/2, Dbl’s big sister, or faciogenital dysplasia 5 (Maldonado et al, 2020). Thus, specific GEFs could activate Rho GTPases, which trigger MLK3-MEK-dependent membrane repair pathways in response to bacterial CDCs.

Here, we tested the hypothesis that the Rac GEF Vav activates MLK3-MEK repair of membrane damage caused by bacterial CDCs. Blocking Vav2, but not other Rac GEFs, nor cdc42 and Rho, halted the MLK3/MEK signaling axis. Moreover, Vav activation accounted for almost all Ca^2+^-dependent repair against CDCs in multiple cell lines. We propose that this additional repair occurs because Vav2 acts upstream of dysferlin and annexins A1, and A2. Our results suggest that Vav2 is an upstream master regulator coordinating multiple membrane repair pathways to protect against CDCs.

## Results

### Rac inhibition sensitizes cells to bacterial PFTs

**Figure S1.**
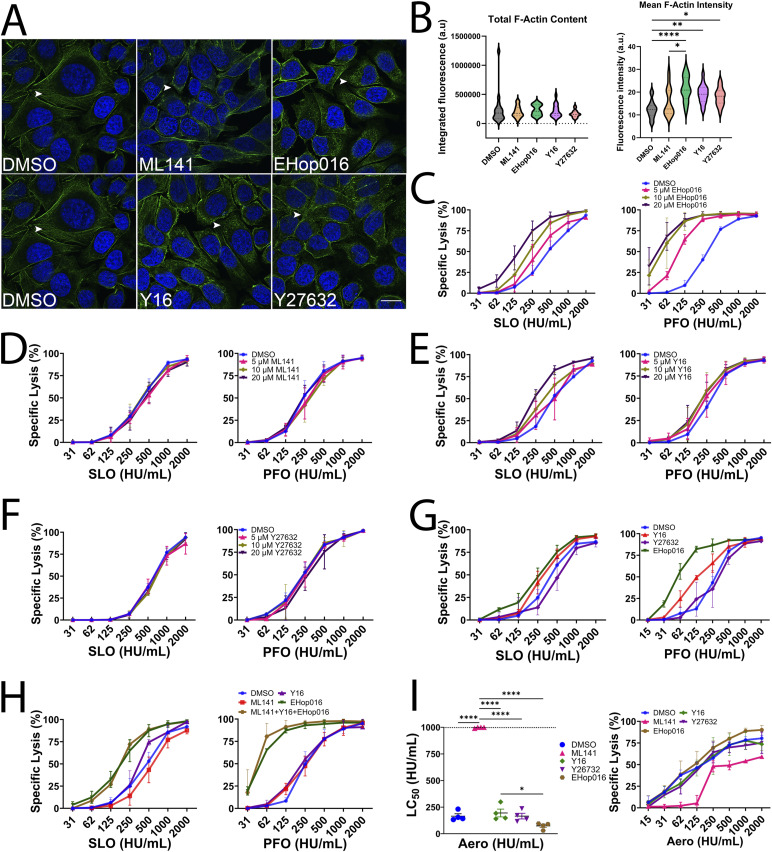
Rac inhibition sensitizes cells to bacterial pore-forming toxins. **(A)** HeLa cells plated on coverslips were serum-starved for 30 min, treated with DMSO, or 20 μM of the indicated inhibitor for 30 min, fixed, stained with phalloidin Alexa flour 488 (green) and DAPI (blue), and imaged by confocal microscopy. Arrowheads point to actin cytoskeleton or its disruption. Micrographs represent three independent experiments. Scale bar = 10 μm. **(B)** Actin content from the micrographs was quantitated to show total and mean F-actin content. For each condition, individual cells were manually outlined on the phalloidin signal (green channel) to define whole cell regions of interest (ROIs). Total F-actin content per cell (left) was quantified as the integrated green channel fluorescence within each ROI. Mean F actin intensity per cell (right) was quantified as the average green channel fluorescence within each ROI (integrated intensity normalized to ROI area). Violin plots show the distribution of per cell values for each condition; individual dots represent single cells, and dashed lines within violins denote the median and interquartile range. **(C, D, E, F, G, H, I)** HeLa cells were pretreated with vehicle (DMSO) or 5, 10, 20 μM (C) EHop016, (D) ML141, (E) Y16, (F) Y27632, or (G–I) 20 μM ML141, Y16, Y27632, and/or EHop016 for 30 min. Cells were then challenged with the indicated concentrations of (C–H) SLO, PFO, or (I) aerolysin for 30 min at 37°C. PI uptake was analyzed by flow cytometry. The LC_50_ was calculated as described in the methods. The dotted line indicates the limit of detection. Points on this line had LC_50_ > 1,000 HU/ml. Graphs show (I) individual experiments with mean ± SEM, or (C–H) the mean ± SEM for n = 5 (C, D, E, [SLO], D, E, [PFO]), n = 4 (C, G, [PFO], G, H, [SLO], I, [Aero]), n = 6 (E, SLO), or n = 3 (H, PFO). The dotted line indicates the limit of detection. Points on this line had LC_50_ > 1,000 HU/ml (I). *P < 0.05, **P < 0.01, ***P < 0.0001 denotes statistical significance using repeated-measures ANOVA between groups with Tukey post-test. SLO, streptolysin O; PFO, perfringolysin O.

**Figure 1.**
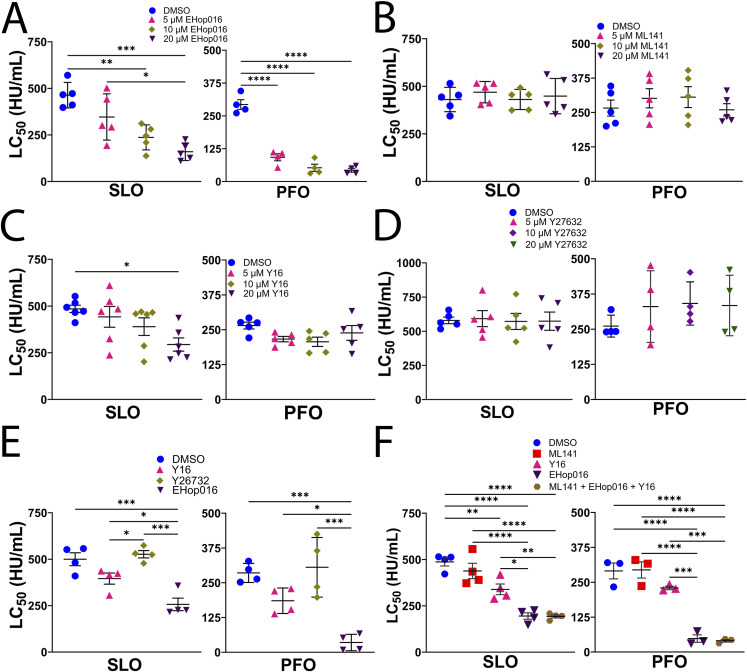
Blocking Rac activation sensitizes cells to bacterial pore-forming toxins. HeLa cells were serum-starved for 30 min, treated with DMSO or 5, 10, or 20 μM **(A)** Rac inhibitor EHop016, **(B)** cdc42 inhibitor ML141, **(C)** Rho inhibitor Y16, **(D)** ROCK inhibitor Y27632, **(E)** 20 μM Y16, Y27632, or EHop016 or **(F)** 20 μM ML141, EHop016, Y16, or all three, and challenged with 31–2,000 HU/ml SLO or PFO for 30 min at 37°C. Propidium iodide (PI) uptake was analyzed by flow cytometry. The LC_50_ was calculated as described in the methods. Graphs show independent experiments and the mean ± SEM, n = 5 (A, B, D [SLO], B, C, [PFO]), n = 4 (A, D, E, [PFO], D, E, F [SLO]), n = 6 (C, SLO), or n = 3 (F, PFO). *P < 0.05, **P < 0.01, ***P < 0.001, ****P < 0.0001 denote statistical significance using repeated-measures ANOVA between groups with Tukey post-test. SLO, streptolysin O; PFO, perfringolysin O.

To determine the contribution of Rho GTPases to repair, we blocked each individually or in combination. We first validated the functional activity of each inhibitor by measuring actin cytoskeleton disruption in HeLa cells treated with each inhibitor. Cdc42, Rho, and Rac inhibitors disrupted actin fiber organization (Fig S1A and B). These data demonstrate that the inhibitors were active. Next, we titrated the inhibitors to determine at what dose, if any, they altered cell sensitivity to CDCs. We pretreated HeLa cells with 5–20 μM of Rac inhibitor EHop016, cdc42 inhibitor ML141, Rho inhibitor Y16 or ROCK inhibitor Y27632, then challenged cells with SLO or PFO. To measure toxin activity, we used hemolytic units (HUs) instead of mass because it normalizes toxin activity across toxin preparations and experiments (Table S1). We fit dose-response curves to a logistic model to determine the lethal concentration of 50% (LC_50_), the toxin dose needed to kill 50% of the cells (Haram et al, 2022), which enables us to compare the impact of each inhibitor. Inhibitors were not toxic to cells unchallenged with CDCs. When challenged with SLO or PFO, Rac inhibition increased the sensitivity of HeLa cells to bacterial toxins by 80–90% compared with vehicle-treated (control) cells (Figs 1A and S1C). In contrast, cdc42 inhibition did not alter cell sensitivity to either toxin compared with DMSO-treated control cells (Figs 1B and S1D). Similarly, Rho inhibition increased sensitivity to SLO at 20 μM but not to PFO (Figs 1C and S1E). Because Rho can signal through ROCK1/2, we tested if ROCK1/2 signaling is involved in SLO sensitivity. ROCK1/2 inhibition did not alter cell sensitivity to either toxin (Figs 1D and S1F). When we compared Rho, Rac, and ROCK1/2 inhibition, the increase in sensitivity to SLO and PFO of Rac-inhibited cells was statistically significant compared with vehicle and other inhibitors (Figs 1E and S1G). We then combined inhibitors to determine any redundancies in CDC sensitivity. We found no increase in sensitivity when other inhibitors were combined with Rac inhibition compared with Rac inhibition alone (Figs 1F and S1H). Therefore, we conclude that the Rac inhibitor EHop016 sensitizes cells to SLO and PFO.

Table S1. Specific activity and protein concentration of toxins used.

Because Rac inhibition sensitizes cells to SLO and PFO, we next determined if Rac was needed for repair against other toxins. The small pore-forming toxin aerolysin is resisted primarily by patch repair instead of microvesicle shedding (Thapa & Keyel, 2023). We challenged Rho, ROCK1/2, or Rac-inhibited HeLa cells with aerolysin. There was a trend towards increased HeLa cell sensitivity to aerolysin upon Rac inhibition (Fig S1I). Interestingly, cdc42 inhibition increased cellular resistance to aerolysin challenge (Fig S1I). Overall, these data suggest that Rac triggers specific repair pathways to protect cells from CDCs.

**Figure 2.**
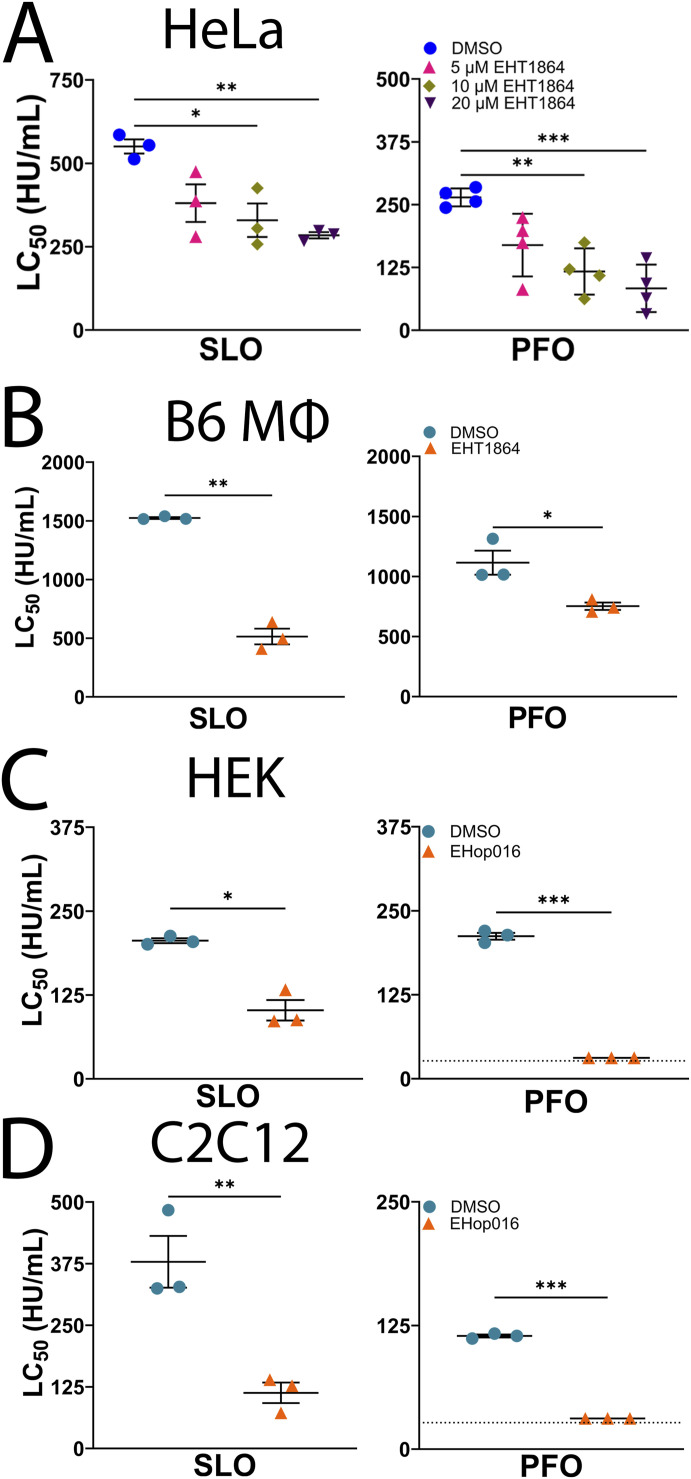
Multiple cell types are susceptible to toxins upon Rac inhibition. **(A)** HeLa, **(B)** bone marrow-derived macrophages (B6 Mϕ), **(C)** HEK, or **(D)** C2C12 cells were serum-starved for 30 min, treated with (A) DMSO or 5, 10, or 20 μM Rac inhibitor EHT1864, DMSO or (B) 20 μM EHT1864, or (C, D) 20 μM EHop016 for 30 min, challenged with SLO or PFO at 37°C for 30 min, and analyzed by flow cytometry for PI uptake. The LC_50_ was calculated as described in the methods. The dotted line indicates the limit of detection. Points on this line had LC_50_ < 15 HU/ml (C, D, PFO). Graphs display independent experiments and the mean ± SEM for n = 3 (A–D) or n = 4 (A, PFO). *P < 0.05, **P < 0.01, ***P < 0.001 denote statistical significance using repeated-measures ANOVA between groups with Tukey post-test. SLO, streptolysin O; PFO, perfringolysin O.

**Figure S2.**
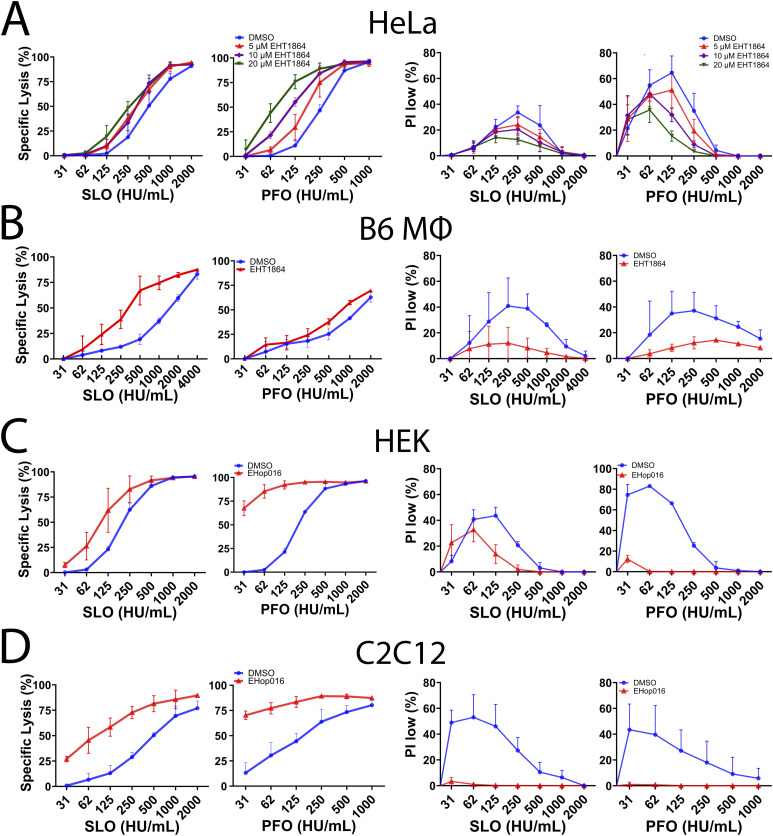
Multiple cell types are sensitive to Rac inhibition. **(A)** HeLa, **(B)** bone marrow-derived macrophages (B6 Mϕ), **(C)** HEK, or **(D)** C2C12 cells were serum-starved for 30 min, treated with DMSO or 5, 10, or 20 μM (A) Rac inhibitor EHT1864, (B) 20 μM EHT1864, or (C and D) 20 μM EHop016 for 30 min, challenged with the indicated concentrations of SLO or PFO, and analyzed by flow cytometry. Specific lysis (PI^high^ population), or transiently repaired cells (PI^low^ are shown). Graphs display the mean ± SEM for n = 3 (A, B, C, D) or n = 4 (A, PFO) independent experiments. SLO, streptolysin O; PFO, perfringolysin O.

To validate the importance of Rac for repair, we examined multiple Rac inhibitors and multiple cell types. We treated HeLa cells with a second Rac inhibitor, EHT1864, before SLO or PFO challenge. We found increased cell sensitivity, similar to EHop016 (Figs 2A and S2A). Next, we evaluated the role of Rac inhibition in membrane repair in other cell types. We inhibited Rac using EHT1864 in primary bone marrow-derived macrophages (BMDMs) from C57BL/6 (B6) mice or using EHop16 in Human Embryonic Kidney (HEK) cells and C2C12 murine myoblasts before CDC challenge. Rac inhibition increased cell death compared with control cells in all cell types (Figs 2B–D and S2B–D). We next compared the transiently permeabilized cell population (Keyel et al, 2011). Rac inhibition reduced the fraction of cells that were transiently repaired and/or shifted the sensitivity to lower concentrations (Fig S2). Therefore, we conclude that multiple mammalian cell types rely on this pathway for repair.

### Vav mediates toxin resistance

**Figure S3.**
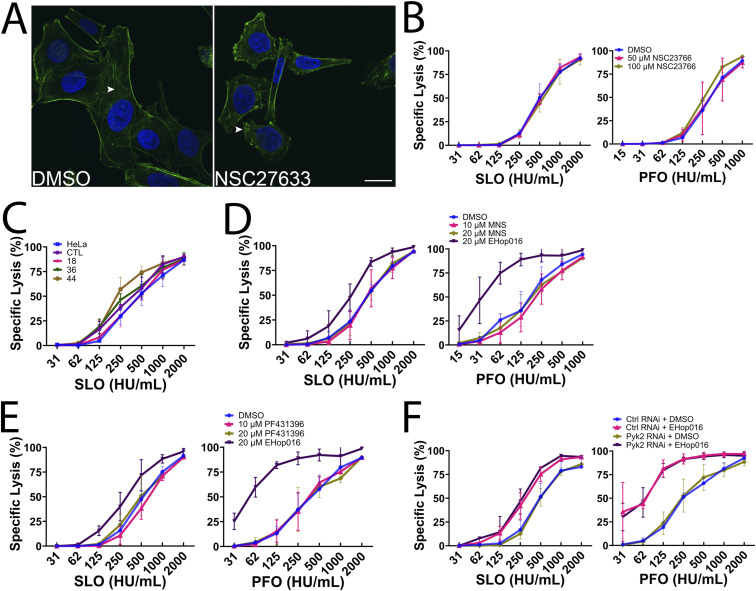
Vav2 promotes membrane repair independent of the tyrosine kinases Src, Syk, focal adhesion kinase, and Pyk2. **(A)** HeLa cells plated on coverslips were serum-starved for 30 min, treated with DMSO or 100 μM NSC23766 for 30 min, fixed, stained with phalloidin Alexa Fluor 488 (green) and DAPI (blue), and imaged by confocal microscopy. Arrowheads point to actin cytoskeleton or its disruption. **(B)** HeLa cells were serum-starved for 30 min, treated with DMSO or 50, 100 μM NSC23766 and challenged with the indicated concentrations of SLO or PFO for 30 min at 37°C. Propidium iodide (PI) uptake was analyzed by flow cytometry. **(C)** Untransfected HeLa cells, CRISPR Control clone (CTL), or Vav2 CRISPR clones (18, 36, and 44) were challenged with the indicated concentrations of SLO for 30 min at 37°C and analyzed as in (B). **(D, E)** HeLa cells were treated as in (B) using (D) 5, 10, or 20 μM Src/Syk inhibitor MNS or (E) 5, 10, 20 μM FAK/Pyk2 inhibitor PF431396. **(F)** Control or Pyk2B-siRNA transfected HeLa cells were treated with DMSO or 20 μM EHop016 for 30 min and then challenged with the indicated concentrations of SLO or PFO for 30 min. Graphs show the mean ± SEM for n = 4 (B, D, SLO) or n = 3 ((C, E, F, [SLO]), (B, D, E, F, [PFO])). Micrographs represent three independent experiments. Scale bar = 10 μm. SLO, streptolysin O; PFO, perfringolysin O.

**Figure 3.**
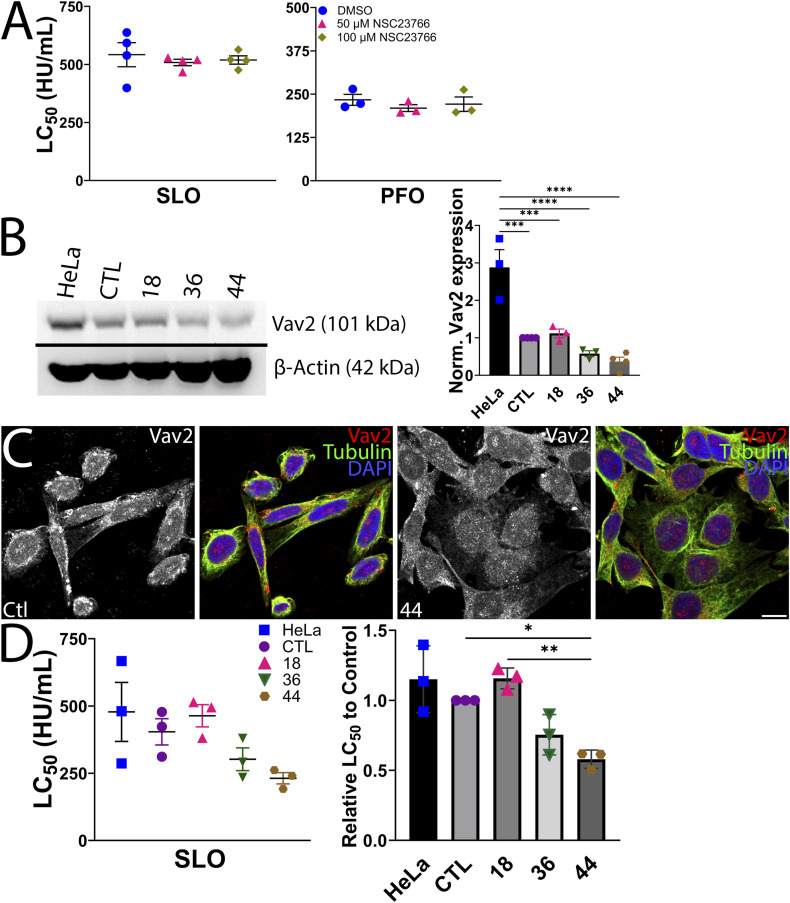
Vav2 promotes membrane repair. **(A)** HeLa cells were serum-starved for 30 min, treated with DMSO or 50 or 100 μM NSC23766 for 30 min, then challenged with 31–2,000 HU/ml SLO or PFO for 30 min at 37°C. PI uptake was analyzed by flow cytometry. The LC_50_ was calculated as described in the methods. **(B, C, D)**. HeLa cells were transfected with negative control guide RNA or guide RNA targeting *Vav2*. Cells were harvested, single cell cloned and expanded. **(B)** Western blot of untransfected HeLa cells, one CRISPR negative control clone (CTL), and three Vav2 clones (18, 36, and 44). Western blots from multiple passages were quantitated and relative Vav2 expression determined. **(C)** CTL or Vav2 CRISPR clone 44 were fixed, permeabilized, and stained with anti-Vav2 (red), anti-tubulin (green) and DAPI (blue). **(D)** Cells were challenged with 31–2,000 HU/ml SLO for 30 min at 37°C. PI uptake was analyzed by flow cytometry. The LC_50_ relative to CTL cells was determined for each experiment. Graphs show independent experiments, and the mean ± SEM for n = 4 (A, SLO) or n = 3 (A PFO, D) independent experiments. The blot and micrographs represent three independent experiments. *P < 0.05, **P < 0.01, ***P <0.001, ****P <0.0001 denote statistical significance using repeated-measures ANOVA between groups with Tukey post-test. SLO, streptolysin O; PFO, perfringolysin O. Source data are available for this figure.

We next narrowed the number of Rac GEFs that could be responsible for Rac activation during membrane repair. Whereas EHT1864 blocks all Rac GEFs, including Vav (Shutes et al, 2007), some Rac inhibitors only block a subset of Rac GEFs. For example, EHop016 inhibits Vav activation of Rac (Montalvo-Ortiz et al, 2012). To determine if other GEFs promote Rac-dependent repair, we blocked Tiam1 and Trio using the inhibitor NSC23766 (Gao et al, 2004). We validated the functional activity of NSC23766 by checking for actin cytoskeleton disruption (Fig S3A). In contrast to Rac inhibition via EHop016, inhibition with NSC23766 failed to increase the sensitivity of HeLa cells to toxin challenge (Figs 3A and S3B). This suggests that Tiam1 and Trio are not involved in activating Rac during repair, but Vav is.

HeLa cells express Vav2 and Vav3 because Vav1 is restricted to hematopoetic cells. Because Vav3 has regulatory roles (Bustelo, 2014), we targeted *Vav2* gene expression using CRISPR. After two attempts at CRISPR, we grew out clones with Vav2 expression reduced ∼67% compared with CRISPR control cells, which was stable across passages (Fig 3B). We examined the heterogeneity of our cells to determine if the residual Vav2 expression was because of mosaic *Vav2* expression or if all cells lost two of the three *Vav2* alleles present in HeLa cells (Landry et al, 2013). We found Vav2 CRISPR cells showed even, residual Vav2 staining (Fig 3C). If the Vav2 cells had mosaic expression, the variance in Vav2 intensity is expected to be distinct from the variance present in control cells. We tested this hypothesis using Levene’s test. None of our three replicates showed unequal variance between control and Vav2 CRISPR cells (Table S2). This suggests we eliminated two of the three *Vav2* alleles, instead of having mosaic expression. Cell sensitivity to SLO increased when Vav2 was >50% reduced in cells (Figs 3D and S3C). Based on these data, we conclude that Vav2 drives membrane repair.

Table S2. Levene’s test on data in Fig 3C.

**Figure 4.**
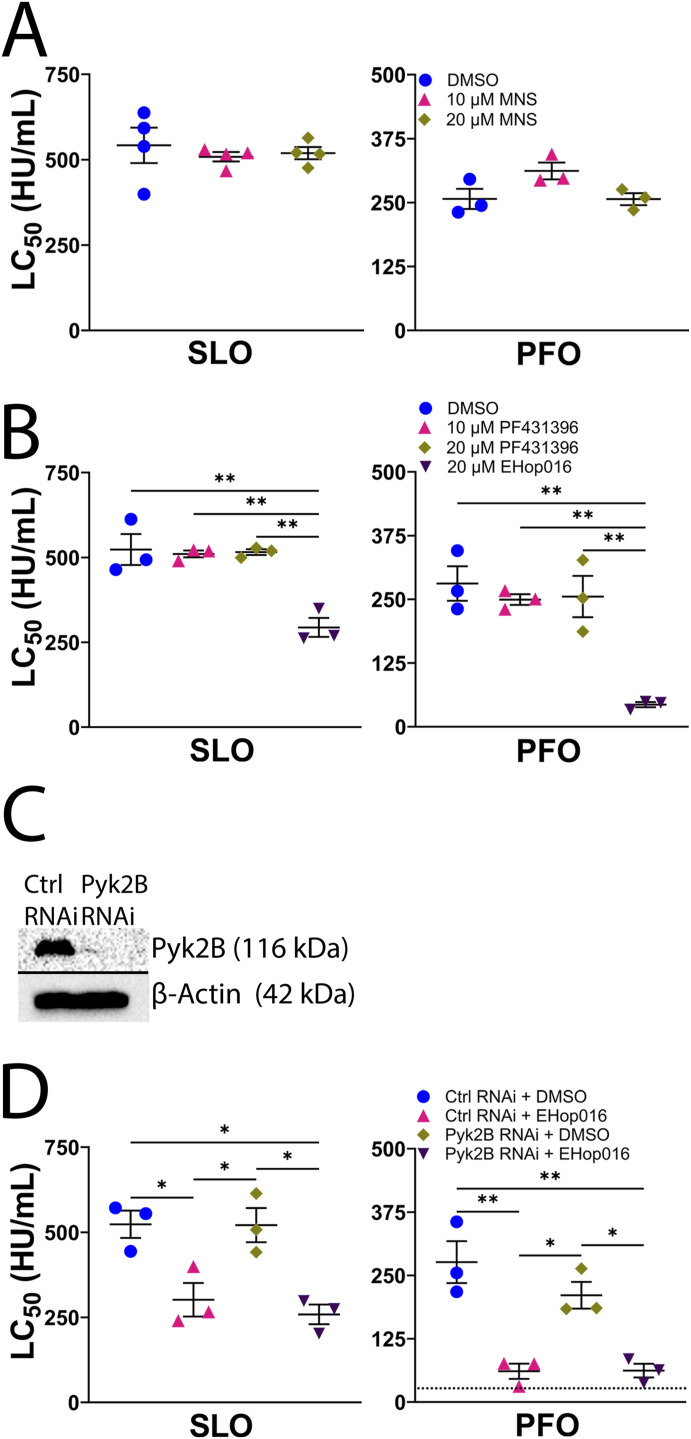
Vav2 promotes membrane repair independent of the tyrosine kinases Src, Syk, focal adhesion kinase, and Pyk2. HeLa cells were serum-starved for 30 min, treated with DMSO or 10 or 20 μM **(A)** Src/Syk inhibitor MNS or **(B)** FAK/Pyk2 inhibitor PF431396 or EHop016 for 30 min, and then challenged with 31–2,000 HU/ml SLO or PFO for 30 min. **(C, D)** HeLa cells were transfected with control (Ctrl) or Pyk2-siRNA for 72 h, and (C) lysed for western blot analysis or (D) serum-starved for 30 min, treated with DMSO or 20 μM EHop016, and challenged with 31–2,000 HU/ml SLO or PFO for 30 min. PI uptake was analyzed by flow cytometry. The LC_50_ was calculated as described in the methods. The dotted line indicates the limit of detection. Points on this line had LC_50_ < 31 HU/ml. Graphs show independent experiments, and the mean ± SEM for n = 4 (A, SLO) or n = 3 (A PFO, B and D) independent experiments. The blot represents three independent experiments. *P < 0.05, **P < 0.01 denote statistical significance using repeated-measures ANOVA between groups with Tukey post-test. SLO, streptolysin O; PFO, perfringolysin O. Source data are available for this figure.

Because Vav2 is a phosphoprotein, we next tested several upstream kinases that could activate Vav2 during membrane repair. We inhibited Src/Syk tyrosine kinases using the inhibitor 3,4-methylenedioxy-β-nitrostyrene (MNS) (Wang et al, 2006) and focal adhesion kinase and Pyk2B with the inhibitor PF431396 (Han et al, 2009). However, neither inhibitor increased CDC sensitivity (Figs 4A and B, and S3D and E). Similarly, siRNA knockdown of Pyk2B failed to increase HeLa cell sensitivity compared with control cells (Figs 4C and D, and S3F). These results suggest that Vav2 promotes membrane repair independent of the tyrosine kinases Src, Syk, focal adhesion kinase, and Pyk2B.

### Vav2 promotes Ca^2+^-dependent repair upstream of MLK3-MEK signaling

**Figure 5.**
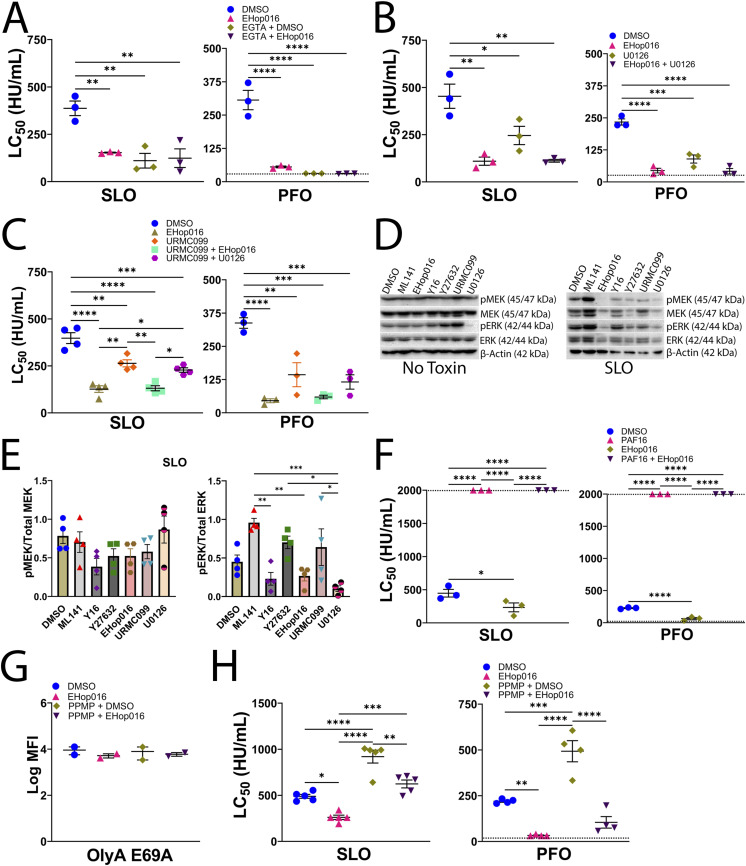
Vav2 promotes Ca^2+^-dependent repair upstream of MLK3-MEK signaling and parallel to ceramide-mediated repair. **(A, B, C)** HeLa cells were serum-starved for 30 min and pretreated with DMSO and/or a combination of: 20 μM Rac inhibitor EHop016, calcium chelator EGTA, 20 μM MEK inhibitor U0126, or 20 μM MLK3 inhibitor URMC099 and challenged with 15–2,000 HU/ml SLO or PFO for 30 min at 37°C. PI uptake was analyzed by flow cytometry. The LC_50_ was calculated as described in the methods. Where indicated, 2 mM EGTA was used instead of 2 mM CaCl_2_. **(D, E)** HeLa cells treated with DMSO or 20 μM ML141, Y16, Y27632, EHop016, or URMC099 were challenged with nothing or sub-lytic SLO (250 HU/ml) for 30 min, and analyzed by western blot for the indicated antibodies. The ratio of phospho-proteins to total protein was calculated. **(F)** HeLa cells were treated and analyzed as in (A) using 20 μM EHop016 and/or MEK activator PAF16. **(G, H)** HeLa cells were treated with 20 μM PPMP for 72 h at 37°C. Then, cells were serum-starved for 30 min at 37°C, treated with DMSO or 20 μM EHop016 for 30 min, and challenged with either (G) 20 μg/ml Ostreolysin A (OlyA) E69A–mCherry, for 5 min, or (H) 31–2,000 HU/ml SLO or PFO for 30 min. Graphs display independent experiments and the mean ± SEM for n = 2 (G), n = 3 (A, B, F, [SLO], A, B, C, F, [PFO]) or n = 4 (C, E, [SLO], E, H, [PFO]) or n = 5 (H, SLO). The western blot represents four independent experiments. The dotted line indicates the limit of detection. Points on this line had LC_50_ < 15 HU/ml (A, [PFO]), LC_50_ < 31 HU/ml (B, [PFO]), or LC_50_ > 2,000 HU/ml (F). *P < 0.05, **P < 0.01, ***P < 0.001, ****P < 0.0001 denote statistical significance using repeated-measures ANOVA between groups with Tukey post-test. SLO, streptolysin O; PFO, perfringolysin O. Source data are available for this figure.

**Figure S4.**
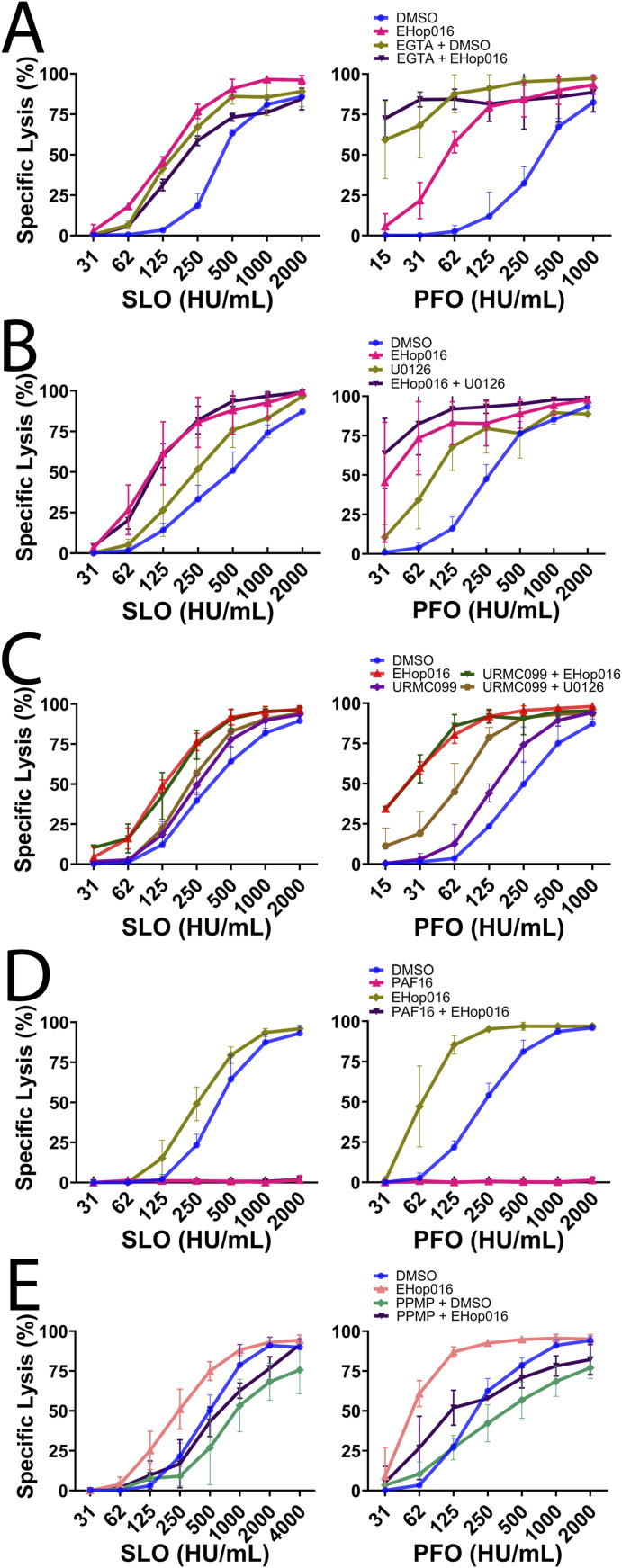
Vav2 promotes Ca^2+^-dependent repair upstream of MLK3-MEK signaling but parallel to ceramide-mediated repair. **(A, B, C, D)** HeLa cells were serum-starved for 30 min and treated with DMSO, 20 μM EHop016, (B–C) U0126, (C) URMC099, and/or (D) 20 μM PAF16 for 30 min and then challenged with the indicated concentrations of SLO or PFO for 30 min. Where indicated, 2 mM EGTA was used instead of 2 mM CaCl_2_. **(E)** HeLa cells were treated with 20 μM PPMP for 72 h at 37°C, then treated with DMSO or 20 μM EHop016, challenged and analyzed as in (A). Graphs display the mean ± SEM for n = 3 (A, B, D, [SLO], A, B, C, D, E, [PFO]) or n = 4 (C, SLO) or n = 5 (E, SLO). SLO, streptolysin O; PFO, perfringolysin O.

To determine if Vav-mediated membrane repair is activated by Ca^2+^ influx, we pretreated HeLa cells with EHop016, with or without Ca^2+^-chelation using EGTA. EGTA alone increased cell sensitivity, consistent with prior results (Keyel et al, 2011; Cooper & McNeil, 2015; Romero et al, 2017; Ray et al, 2022; Thapa & Keyel, 2023) (Figs 5A and S4A). When EGTA was combined with Vav inhibition, no additive effects were observed, suggesting that Vav acts in the Ca^2+^-dependent pathways (Figs 5A and S4A). Thus, Vav-dependent repair is activated after Ca^2+^ influx.

We next determined if Vav2 acts upstream of MLK3-MEK signaling. We tested this hypothesis by blocking MEK with the previously characterized MEK inhibitor U0126 (Ray et al, 2022), and/or Vav activity with EHop016 in HeLa cells before toxin challenge. After toxin challenge, both inhibitors reduced cell survival (Figs 5B and S4B). However, inhibition of both Vav and MEK did not further exacerbate toxin-induced cell death compared with Vav inhibition alone (Figs 5B and S4B). These data suggest that Vav and MEK are in the same repair pathway. Next, we blocked Vav activity and/or MLK3 and compared the inhibition with that achieved by blocking both MLK3 and MEK. Upon toxin challenge, Vav inhibition blocked more repair to SLO and PFO than MLK3 inhibition alone or MLK3-MEK inhibition combined (Figs 5C and S4C). These data suggest that Vav acts upstream of the MLK3-MEK signaling pathway during membrane repair.

To confirm that Vav acts upstream of MLK3 and MEK, we measured the phosphorylation of MEK and ERK after Vav inhibition. Whereas repair is ERK-independent (Ray et al, 2022), ERK phosphorylation is a readout for MEK activity. Relative to control cells, at baseline, ERK and MEK phosphorylation were unchanged for all inhibitors except MEK blockade, consistent with the ability of Raf and other kinases to activate MEK and ERK (Fig 5D and E). However, upon challenge with a sub-lytic dose of SLO, inhibition of Rho, Rac, or MEK all decreased ERK phosphorylation, whereas cdc42 inhibition increased ERK phosphorylation (Fig 5D and E). If MEK acts downstream of Vav during repair, we predict forced MEK activation would override Vav inhibition and protect cells from CDCs. We tested this hypothesis by pretreating HeLa cells with EHop016 and/or the MEK activator platelet-activating factor 16 (PAF16) before and during the toxin challenge. As expected, Vav inhibition increased cell sensitivity to toxins (Figs 5F and S4D). In contrast, MEK stimulation using PAF16 protected HeLa cells from SLO or PFO, regardless of Vav inhibition (Figs 5F and S4D). Based on these data, we conclude that Vav acts upstream of MLK3 and MEK signaling during membrane repair.

### Vav and ceramide-mediated repair are parallel

Because Vav acts upstream of MLK3 and confers greater protection to cells, we tested other Ca^2+^-dependent repair pathways to determine if they act in the same pathway as Vav or a parallel pathway to Vav. Ceramide contributes to repair (Schoenauer et al, 2019; Ray et al, 2022; Haram et al, 2023), via a parallel pathway to MEK (Ray et al, 2022). We tested the hypothesis that Vav is involved in ceramide-driven repair. To elevate ceramide without sphingomyelin depletion, we used d-threo-1-phenyl-2-hexadecanoylamino-3-morpholino-1-propanol HCl (PPMP) for 72 h to induce accumulation of ceramide in cells (Dany & Ogretmen, 2015). Neither PPMP treatment nor Vav inhibition altered total surface sphingomyelin levels (Fig 5G). When HeLa cells were challenged with toxins, PPMP protected cells (Figs 5H and S4E), as previously observed (Ray et al, 2022). Whereas EHop016 reduced the LC_50_ of PPMP-treated cells, it failed to make cells as sensitive as Vav inhibition alone (Figs 5H and S4E). Because Vav inhibition partly overcame ceramide protection, we conclude that Vav-driven and ceramide-mediated repair are parallel repair pathways.

### Vav may regulate dysferlin and annexin-mediated repair

**Figure 6.**
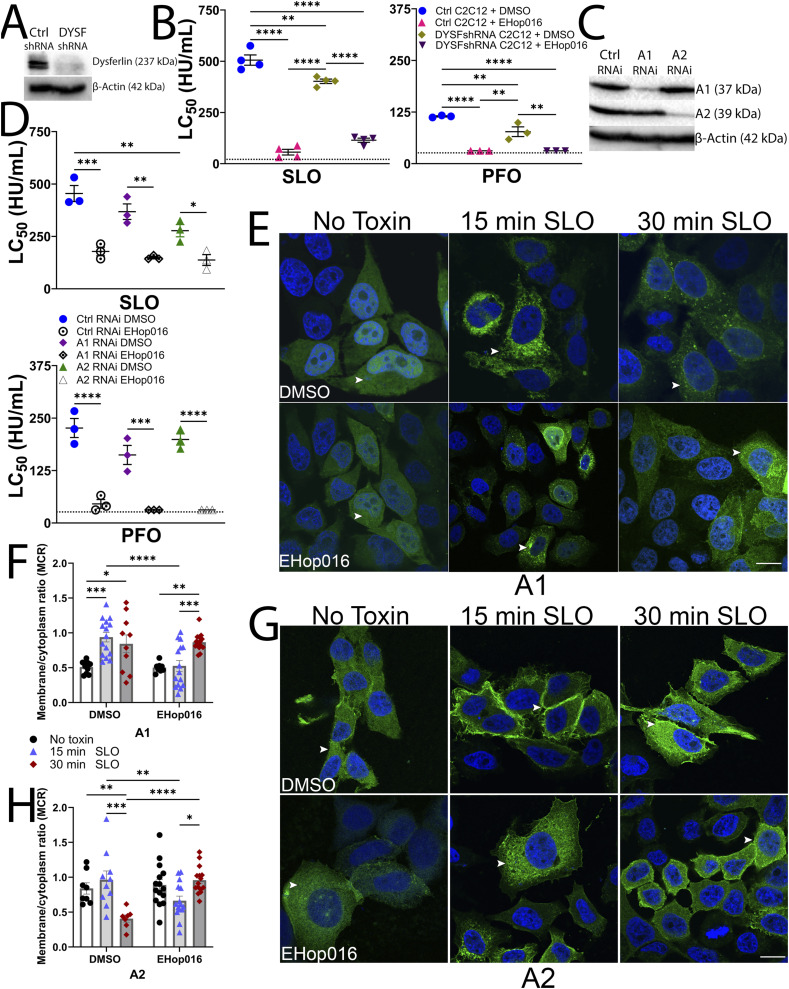
Vav regulates dysferlin and annexin-mediated repair. **(A, B)** Control shRNA or dysferlin shRNA C2C12 cells were (A) lysed for western blot analysis, or (B) serum-starved for 30 min, treated with DMSO or 20 μM EHop016, and challenged with 15–2,000 HU/ml SLO or PFO for 30 min. PI uptake was analyzed by flow cytometry. **(C, D)** HeLa cells transfected with control (Ctrl), annexin A1 (A1), or annexin A2 (A2) siRNA for 72 h were (C) lysed for western blot analysis or (D) serum-starved for 30 min, pre-treated with DMSO or 20 μM EHop016 for 30 min, and challenged with 31–2,000 HU/ml SLO or PFO for 30 min. PI uptake was analyzed by flow cytometry. The LC_50_ was calculated as described in the methods. HeLa cells plated on coverslips were transfected with (E, F) A1-YFP or (G, H) A2-GFP, serum-starved for 30 min, pre-treated with DMSO or 20 μM EHop016 for 30 min, and challenged with sub-lytic SLO (250 HU/ml) for 0, 15, or 30 min at 37°C. **(E, G)** Cells were fixed, stained with DAPI, and imaged. Arrowheads show membrane versus cytoplasmic annexins. **(F, H)** Translocation of annexins to the membrane was quantified. Graphs show independent experiments and the mean ± SEM for n = 4 (B, SLO) or n = 3 (D [SLO], B, D, [PFO]) or individual cells and the mean ± SD from three independent experiments (F and H). The dotted line indicates the limit of detection. Points on this line had LC_50_ < 15 (B, D [PFO]) or 31 HU/ml (B [SLO]). The blots represent 3 independent experiments. Micrographs represent three independent experiments. Scale bar = 10 μm.*P < 0.05, **P < 0.01, ***P < 0.001, ****P < 0.0001 denote statistical significance using repeated-measures ANOVA between groups with Tukey post-test. SLO, streptolysin O; PFO, perfringolysin O. Source data are available for this figure.

**Figure S5.**
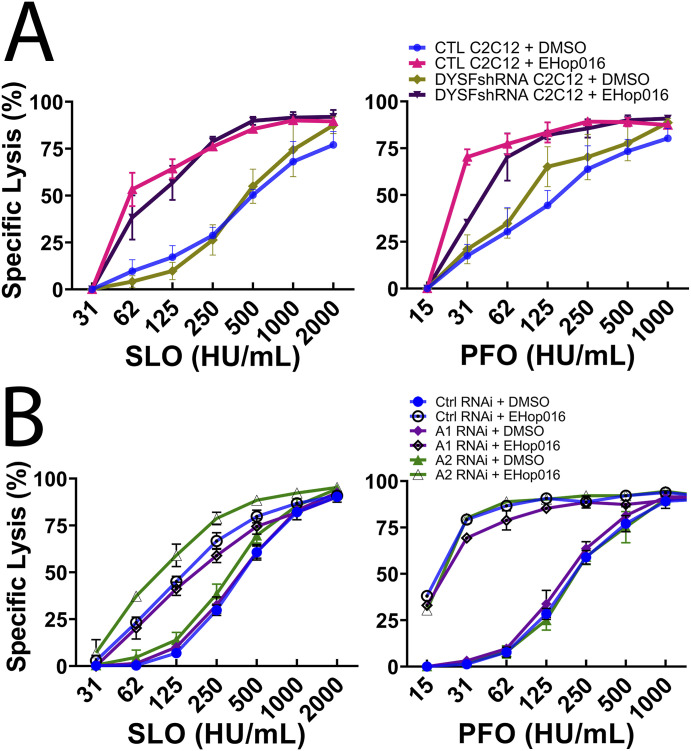
Vav regulates dysferlin and annexin-mediated repair. **(A, B)** Dysferlin shRNA or control shRNA C2C12 or (B) HeLa cells transfected with control (Ctrl), annexin A1 (A1), or annexin A2 (A2) siRNA were serum-starved for 30 min, treated with DMSO or 20 μM EHop016 for 30 min and then challenged with the indicated concentrations of SLO or PFO for 30 min. PI uptake was analyzed by flow cytometry. Graphs show the mean ± SEM for n = 4 (A, [SLO]), or n = 3 (B, [SLO] A, B, [PFO]). SLO, streptolysin O; PFO, perfringolysin O.

Next, we investigated the role of Vav in patch repair by determining its role in dysferlin-mediated repair. We inhibited Vav in C2C12 myoblasts stably transfected with control or dysferlin shRNA before the toxin challenge. Dysferlin knockdown was confirmed by western blot (Fig 6A). Consistent with previous results (Thapa & Keyel, 2023), dysferlin loss increased cell sensitivity to CDCs (Figs 6B and S5A). Vav inhibition increased the CDC sensitivity of cells to a similar extent, regardless of dysferlin expression (Fig S5A). When the cells were too sensitive to SLO during Vav inhibition to calculate LC_50_, we compared the full dose-response curves to determine additive or non-additive effects (Fig S5A). We interpret these data to suggest that Vav regulates dysferlin-mediated repair to toxin damage.

Next, we tested the involvement of Vav in annexin-mediated clogging. We depleted annexins A1 (A1) and A2 (A2) by siRNA and confirmed knockdown by western blot (Fig 6C). We then challenged these annexin-depleted cells with SLO or PFO in the presence or absence of Vav inhibition. Consistent with prior results (McNeil et al, 2006; Swaggart et al, 2014; Thapa & Keyel, 2023), we found that knockdown of each annexin alone increased cell sensitivity to CDCs compared with control cells (Figs 6D and S5B). However, A1 or A2 depletion failed to exacerbate cell death beyond that caused by Vav inhibition after CDC challenge (Fig S5B). These data suggest that Vav acts upstream of multiple annexins during repair against bacterial CDCs.

We next examined the rate of annexin binding to the membrane during Rac inhibition. Our prior results showed that A2 membrane recruitment took over 20 min when MEK was blocked, but A1 had accelerated membrane recruitment (Ray et al, 2022). To determine if A1 and A2 both had retarded recruitment or if recruitment was normal, we measured annexin recruitment in vehicle-treated or Vav-inhibited cells at 15 and 30 min post-SLO challenge by high-resolution confocal microscopy. Both annexins were recruited to the membrane from the cytosol within 15 min in control cells (Fig 6E–H). In contrast, Vav inhibition delayed A1 and A2 recruitment with limited recruitment to the membrane even after 30 min (Fig 6E–H). Based on these data, we conclude that Vav signaling is needed to recruit A1 and A2 during membrane repair.

## Discussion

Here, we found that Vav2 serves as an upstream activator of membrane repair in response to CDCs in multiple mammalian cell types. Vav2 acted in multiple Ca^2+^-dependent repair pathways, accounting for ∼90% of Ca^2+^-dependent repair. Consistent with this large contribution to membrane repair, Vav2 coordinated microvesicle shedding via the MLK3-MEK pathway, patch repair via dysferlin, and clogging via annexin recruitment. However, we found that ceramide-mediated protection was a parallel pathway to Vav. This suggests that Vav activation is an early event during membrane repair because it serves as a master regulator in response to bacterial CDCs.

We identified the Rac GEF that activates multiple, distinct membrane repair pathways. Our findings provide new context for prior work showing that Rac1 is activated by the CDCs pneumolysin and listeriolysin O, or laser wounding, and remodels actin upon activation (Togo & Steinhardt, 2004; Iliev et al, 2007; Lam et al, 2018). Whereas cdc42 and Rho activation are needed for repair from laser wounding in *Xenopus* oocytes and *Drosophila* embryos (Benink & Bement, 2005; Abreu-Blanco et al, 2014; Moe et al, 2021), we did not observe that for SLO or PFO. This is consistent with prior findings that listeriolysin O damage triggered Rac (Lam et al, 2018). Because actin remodeling is a well-established outcome of Rac signaling, we did not focus on actin remodeling in this study. However, our findings suggest that Rac signaling has the capacity to mediate repair via multiple mechanisms beyond actin remodeling.

Distinct from prior findings focused on actin remodeling during repair, we now connect Rac activation by Vav2 to other established downstream membrane repair pathways, including MEK-dependent repair, annexins, and dysferlin activation. Future work is needed to determine if Rac1 and Rac3 are redundant and how Rac activates these repair pathways. Because Rac binds directly to the crib domain of MLK3 (Burbelo et al, 1995), it could regulate MEK-dependent repair by directly activating MLK3. The mechanism by which Rac promotes dysferlin or A1 recruitment may be less direct, or Vav2 may activate them via Rac-independent mechanisms (Bustelo, 2014). Thus, our work places Rac in a central role in directing the membrane repair response to bacterial CDCs beyond its involvement in the actin cytoskeleton.

The role of other Rho GTPases remains less clear. Prior work found that Rho mediated Ca^2+^-independent repair (Iliev et al, 2007) and can activate non-muscle myosin II (Togo & Steinhardt, 2004). In contrast, we found that Rho sensitized cells only to SLO. Future work is needed to determine if the differences between SLO and PFO are because of membrane binding, and how this pathway integrates into the larger picture of repair. Based on our results, Rho does not appear to orchestrate a universal Ca^2+^-dependent repair mechanism.

Our work had some limitations. Whereas our data do not support a role for cdc42 in membrane repair or for Rho in repair responding to PFO, we did not rule out activation of these proteins. These proteins could be activated but not necessary for cell survival. We used inhibitors because Rho GTPase inhibitors are well-characterized and carry the greatest translational capacity, but we did not use siRNA for Rho GTPases or GEFs. Our CRISPR cells targeted two of the three Vav2 alleles, leaving residual Vav2 expression that protected cells. Alternatively, there could be clone-specific effects. We did not perform any gain-of-function experiments with Vav2, annexins, or dysferlin. Our analysis focused on SLO and PFO as representative CDCs instead of other CDCs. There is variability in toxin assays, which can complicate interpretation. We did not use a bacterial model for damage to avoid complications from non-toxin factors. Despite these limitations, we provide evidence supporting a sweeping role for Vav activation of Rac in membrane repair to bacterial CDCs.

This work opened new horizons for future study. Identification of the upstream activator for Vav2 and how it connects to Ca^2+^-influx remains to be determined. The mechanisms by which Vav2 regulates A1 and dysferlin remain to be determined. If Vav1 is redundant with Vav2 and if Vav3 antagonizes repair remains to be determined. Another new avenue of research is the extent to which other pathways we were unable to test, like Endosomal Sorting Complex Required for Transport–mediated shedding or other annexins, are controlled by Vav2. Whereas Vav activation coordinates repair against multiple bacterial toxins, Vav may also coordinate resistance to mammalian PFTs and other toxin families. Overall, our results provide an integrated mechanism of membrane repair and suggest that triggering Vav-activated repair pathways could provide a new approach to treating necrotizing soft tissue infections.

## Materials and Methods

### Reagents

Unless otherwise noted, all reagents were from Thermo Fisher Scientific. Propidium iodide (PI) (Cat# P4170-100MG) and DAPI (Cat# D9542) were from Sigma-Aldrich. MEK inhibitor U0126 was from Tocris (Cat# 1144) or Cell Signaling Technology (Cat# 9903S). MLK inhibitor URMC-099 (Cat# 19147) and DL-threo-PPMP (hydrochloride) (Cat# 17236) were from Cayman Chemicals. MEK activator PAF16 (Cat# 2940), Rac inhibitors EHT 1864 (Cat# 3872), NSC23766 (Cat# 2161), and the Src/Syk inhibitor MNS (Cat# 2877) were from Tocris Bioscience. Rho Inhibitor-Y16 (Cat# HY-12649) and ROCK1/2 inhibitor, Y-27632 dihydrochloride (Cat# HY-10583), were from MedChemExpress (NJ, USA). Rac inhibitor EHop016 (Cat# S7319), cdc42 inhibitor ML141 (Cat# S7686), and FAK/Pyk2b inhibitor PF431396 (Cat# S7644) were from Selleckchem. Negative control siRNA (Cat# 462001), Silencer pre-designed siRNAs for annexin A1 (Assay ID: 146988) and A2 (Assay ID: 147285) were from Ambion. PYK2/PTK2B DsiRNA was from Integrated DNA Technologies. Anti-MEK (9122L), anti–phospho–MEK-[Ser217/Ser221] (9121S), anti-ERK p44/42 (9102S), and anti–phospho–ERK [Thr202/Tyr404] (9101S) antibodies were from Cell Signaling Technologies. Anti-Vav2 (Cat# 21924-1AP) was from Proteintech. Anti-β-actin AC-15 mAb (Cat# GTX26276) was from GeneTex. Anti-tubulin E7 mAb was from the Developmental Studies Hybridoma Bank, created by the NICHD of the NIH and maintained at the University of Iowa, Department of Biology. Goat anti-mouse (711-035-151) and anti-rabbit (711-035-152) horseradish peroxidase (HRP)–conjugated antibodies were from Jackson ImmunoResearch.

### Plasmids

The pBAD-gIII plasmid encoding His-tagged SLO lacking the lone Cys (C530A) codon-optimized for *Escherichia coli* expression was previously described (Ray et al, 2022). Cys-less, His-tagged PFO in pET22 (Shepard et al, 1998) was a gift from Rodney Tweten (University of Oklahoma Health Sciences Center, Oklahoma City, OK, USA). Pig annexin A1 fused to YFP was a gift from Annette Draeger (University of Bern, Bern, Switzerland) (Babiychuk et al, 2009). Annexin A2-GFP was a gift from Volker Gerke & Ursula Rescher (Addgene plasmid #107196) (Rescher et al, 2000). OlyA fused to mCherry in pET21c(+) was a gift from Kristina. Sepčić (University of Ljubljana, Ljubljana, Slovenia) (Skocaj et al, 2014). The mCherry-OlyA E69A, which removes the cholesterol requirement for OlyA binding to sphingomyelin, was previously generated (Ray et al, 2022).

### Mice

All experimental mice were housed and maintained in accordance with Texas Tech University Institutional Animal Care and Use Committee (TTU IACUC) standards, adhering to the Guide for the Care and Use of Laboratory Animals (eighth edition, NRC 2011). TTU IACUC approved mouse use for all mouse experimental procedures. C57BL/6 mice were purchased from the Jackson Laboratory (stock #000664). BMDMs were prepared using mice of both sexes aged 6–15 wk as described below. No sex effects were observed. Mice were euthanized by asphyxiation through the controlled flow of pure CO_2_, followed by cervical dislocation.

### Recombinant toxins

Toxins were purified as previously described (Ray et al, 2022; Thapa & Keyel, 2023). Briefly, toxins were induced with 0.2% arabinose (SLO) or 0.2 mM isopropyl-β-d-thiogalactopyranoside (PFO, aerolysin) for 3 h at room temperature, followed by purification using Nickel–NTA resin. Protein concentration was measured by Bradford assay, and hemolytic activity was determined as previously described (Romero et al, 2017) using human red blood cells (Zen-Bio, Research Triangle Park). One HU is the amount of toxin needed to lyse 50% of a 2% human red blood cell solution in 30 min at 37°C in PBS with 2 mM CaCl_2_, 0.3% bovine serum albumin, and 10 mM Hepes (pH 7.4) (Keyel et al, 2012). HUs per milliliter (HU/ml) were used to normalize toxin activities and achieve coherent cytotoxicity across toxin preparations (Table S1).

### Cell culture

HeLa cells (ATCC CCL-2) and HEK-293 (ATCC CRL-1573) were cultured in Dulbecco’s modified Eagle’s medium (DMEM; Corning) supplemented with 10% Equafetal bovine serum (Atlas Biologicals), 1× l-glutamine, 1× penicillin and streptomycin (D10). B6 BMDMs were isolated from bone marrow and cultured as previously described (Romero et al, 2017; Ray et al, 2022). Briefly, BMDMs were differentiated for 7–21 d in DMEM supplemented with 30% L929 cell supernatant, 20% fetal calf serum (VWR Seradigm), 1 mM sodium pyruvate, and 1× L-glutamine. C2C12 myoblasts stably expressing control (ATCC CRL-3419) or Dysf shRNA (ATCC CRL-3418) were cultured in D10 plus 2 μg/ml puromycin. Cells were maintained at 37°C in 5% CO_2_.

### Transfection

For siRNA knockdowns, HeLa cells were plated at a density of 2 × 10^5^ cells in six-well plates. After 24 h, these cells were transfected with 20 nM control, Pyk2, annexin A1, or A2 siRNAs using Lipofectamine 2000 in Opti-MEM for 48–72 h before assays.

For plasmid transfections, HeLa cells were plated at 2 × 10^5^ cells per 35-mm glass-bottom dish, 35-mm dish, or per well of a six-well plate. They were then transfected with 500 ng of annexin A1-YFP or 750 ng of annexin A2-GFP using Lipofectamine 2000 in Opti-MEM 2 d before imaging. The D10 was replaced on the day after transfection. Transfection efficiency for each construct ranged from 50% to 70% across experiments.

### CRISPR knockout of Vav2

HeLa cells were plated at 2.5 × 10^5^ cells in six-well plates. After 24 h, these cells were transfected with either negative control guide RNA (Fisher catalog A35526), or guide RNA targeting *Vav2* (Fisher catalog CRISPR957573_SGM, targeting CGC​ACA​TCG​AAG​AGG​TCA​AA with PAM sequence GGG), along with 2.5 μg TrueCut Cas9 (Cat A36497) using CrisprMax in Opti-MEM. After 4 d, cells were harvested and plated at 0.6 cells/well in 96-well plates for single cell cloning. Remaining cells were assayed for CRISPR efficiency using the GeneArt Genomic Cleavage Detection kit per manufacturer’s instructions. Wells with single colonies were expanded and screened by western blot to determine the extent of Vav2 expression. Control clones and clones with reduced Vav2 expression were then assayed for cytotoxicity, Vav2 expression, and cryopreserved.

### Cytotoxicity assays

Cytotoxicity assays were performed as previously described and validated (Keyel et al, 2011; Ray et al, 2018; Ray et al, 2022; Thapa & Keyel, 2023), with the following modifications. 1 × 10^6^ HeLa cells were plated in 60 mm dishes and incubated at 37°C and 5% CO_2_ incubator for 36–48 h before the cytotoxicity assay. At 80–95% confluency, media were aspirated, cells were washed with 1X PBS and serum-starved in DMEM for 30 min at 37°C. Cells were then treated with 20 μM inhibitors unless otherwise noted before toxin challenge. DMSO was used as the vehicle control, ML141 inhibits cdc42, Y16 inhibits Rho, Y27632 inhibits ROCK1/2, URMC099 inhibits MLK3, U0126 inhibits MEK, EGTA chelates Ca^2+^, EHop016, EHT1864, and NSC23766 inhibit Rac, PF431396 inhibits FAK and Pyk2b, and MNS inhibits Src and Syk. Cells were incubated with inhibitors in DMEM at 37°C, 5% CO_2_ for 30 min before toxin challenge. Then cells were harvested, and 1 × 10^5^ pretreated cells were challenged in suspension with 31–2,000 HU/ml toxin for 30 min at 37°C in RPMI supplemented with 2 mM CaCl_2_ (RC) and 20 μg/ml propidium iodide (PI) in the continued presence of inhibitors or DMSO. Cells were analyzed on a four-laser Attune Nxt flow cytometer. Debris was gated out, and the percentage of single cells with high dye (PI) fluorescence (2 to 3 log shift) (dye high) was quantified. This assay shows membrane integrity after 30 min. Whereas this is fundamentally a membrane integrity assay, we previously showed that using this gating and selecting “dye high” populations accurately reports dead cells (Keyel et al, 2011; Ray et al, 2018). Transient permeabilization can be measured by tracking “dye low” populations. We calculated % specific lysis as follows:

$$\%\;\text{Specific}\;\text{Lysis}=\left({\%\;\text{Dye}\;\text{High}}^{\mathrm{Exp}}-{\%\;\text{Dye}\;\text{High}}^{\text{Ctl}}\right)\;/\;\left(100\;-{\%\;\text{Dye}\;\text{High}}^{\text{Ctl}}\right)\times 100$$ (1)

The toxin dose required to kill 50% of cells was defined as the LC_50_. The LC_50_ was determined by logistic modeling using Excel (Microsoft) as previously described (Haram et al, 2022). The logistic model used was:

$$y=L/\left(1+e^{-k*\left(x-c\right)}\right)$$ (2)

where x is the toxin concentration, y is the % specific lysis, L is maximal specific lysis, c is the toxin concentration at which y is 50% of L, and k measures the steepness of the sigmoidal curve.

### SDS-PAGE and immunoblotting

We performed SDS-PAGE as previously described (Ray et al, 2022; Thapa & Keyel, 2023). Briefly, cells were spun down and washed thrice with 1X PBS before resuspension in 95°C 1X SDS sample buffer, incubation at 95°C for 5 min and sonication. Samples were resolved on 10% polyacrylamide gels and transferred to nitrocellulose in an ice bath with transfer buffer at 110 V for 90 min. Blots were blocked using 5% skim milk in Tris-buffered saline with Tween for 2 h. Portions of the blots were incubated for 2 h at room temperature with any of the following primary antibodies diluted in 1% skim milk in Tris-buffered saline with Tween: CPTC-A1–3 anti-annexin A1 (1:250) mAb, anti-Annexin A2 (1:1,000) mAb, AC-15 anti-β-actin (1:5,000) mAb, rabbit polyclonal antibodies: anti-MEK, anti–phospho-MEK [Ser217/221], anti-ERK p44/42, anti–phospho-ERK [Thr202/Tyr404] at 1:1,000 dilution each, and anti-PYK2 (1:250). After washing, blots were incubated with HRP-conjugated anti-mouse or anti-rabbit immunoglobulin G antibodies (1:10,000) diluted in 1% skim milk and developed with enhanced chemiluminescence (ECL): 0.01% H_2_O_2_ (Walmart), 0.2 mM p-Coumaric acid (Sigma-Aldrich), 1.25 mM luminol (Sigma-Aldrich) in 0.1 M Tris (pH 8.4). Blots from at least three independent experiments were quantified by measuring and comparing band intensities using Photoshop (Adobe). Complete western blots are included as Figure Source Data.

### Immunofluorescence

Immunofluorescence was performed as previously described (Thapa & Keyel, 2023). HeLa cells were plated on coverslips. Cells were washed twice in 1X PBS, fixed in 2% paraformaldehyde for 15 min, washed, permeabilized and blocked with 0.2% saponin, 10% goat serum in PBS, stained with phalloidin conjugated to Alexa Fluor 488 (1:40) for 1 h, washed, DAPI stained, washed, and mounted on slides in Gelvatol. For Vav2 expression, anti-Vav2 (1:200) and anti-tubulin (1:10) were used, with anti-mouse IgG conjugated to Alexa Fluor 488 (1:500) and anti-rabbit IgG conjugated to Alexa Fluor 568 (1:500) as secondary antibodies. Transfected cells were fixed and DAPI stained as described above. Cells were imaged on a Fluoview 3000 confocal microscope (Olympus) with a 60×, 1.42 NA oil-immersion objective. Images were processed using ImageJ (NIH, Bethesda, MD, USA). To quantitate F-actin, individual cells were manually outlined on the phalloidin signal (green channel) to define whole cell regions of interest (ROIs). Total F-actin content per cell was quantified as the integrated green channel fluorescence within each ROI. Mean F-actin intensity per cell was quantified as the average green channel fluorescence within each ROI (integrated intensity normalized to ROI area).

### Statistics

GraphPad Prism 10.0.3 was used for statistical analysis. Statistical significance was determined by one-way analysis of variance (ANOVA) or repeated-measures ANOVA with Tukey post hoc testing. *P* < 0.05 was statistically significant.

## Supplementary Material

Reviewer comments

## Data Availability

All data are in the main text or supplementary materials.
