# Supplementary material for: Vav2 is a master regulator of repair against bacterial pore-forming toxins
Source: Life Sci Alliance. 2026 Jul 9;9(9):e202603633. doi: 10.26508/lsa.202603633 (PMC13351264; doi:10.26508/lsa.202603633)
Supplement: Supplementary file 1 [file LSA-2026-03633_TableS1.docx]

**Supplementary Table S1. Specific activity and protein concentration of toxins used.**

| **Toxin** | **Figure used** | **Hemolytic activity (HU/mL)** | **Protein conc (mg/mL)** | **Specific activity (HU/mg)** |
| --- | --- | --- | --- | --- |
| SLO WT | 1A-D, 2A-D, S1B-H, S2A-D | 1.2 × 10^6^ | 2.7 | 4.4 ×10^6^ |
| SLO WT | 3A, 4A-B, 4D, 5A-F, S3B-E, S4, S5A | 2.0 × 10^6^ | 5.3 | 3.8 ×10^5^ |
| SLO WT | 1E-F, 6B, 6D-F, S5A-B | 4.8 × 10^6^ | 2.7 | 1.8 ×10^6^ |
| PFO WT | 1A-F, 2A-D, 3A, 4A-B, 4D, 5A-C, 5F, 5H, 6B, 6D, S1-4A-D | 2.0 × 10^6^ | 5.6 | 3.6 ×10^5^ |
| PFO WT | 1E-G, S4E, S5A-B | 6.4 × 10^6^ | 7.3 | 8.8 ×10^5^ |
| Aerolysin WT | S1I | 4.0 × 10^5^ | 2 | 2.0 ×10^5^ |
