# Supplementary figures and images for: Vav2 is a master regulator of repair against bacterial pore-forming toxins
Source: Life Sci Alliance. 2026 Jul 9;9(9):e202603633. doi: 10.26508/lsa.202603633 (PMC13351264; doi:10.26508/lsa.202603633)

**Figure 3B**

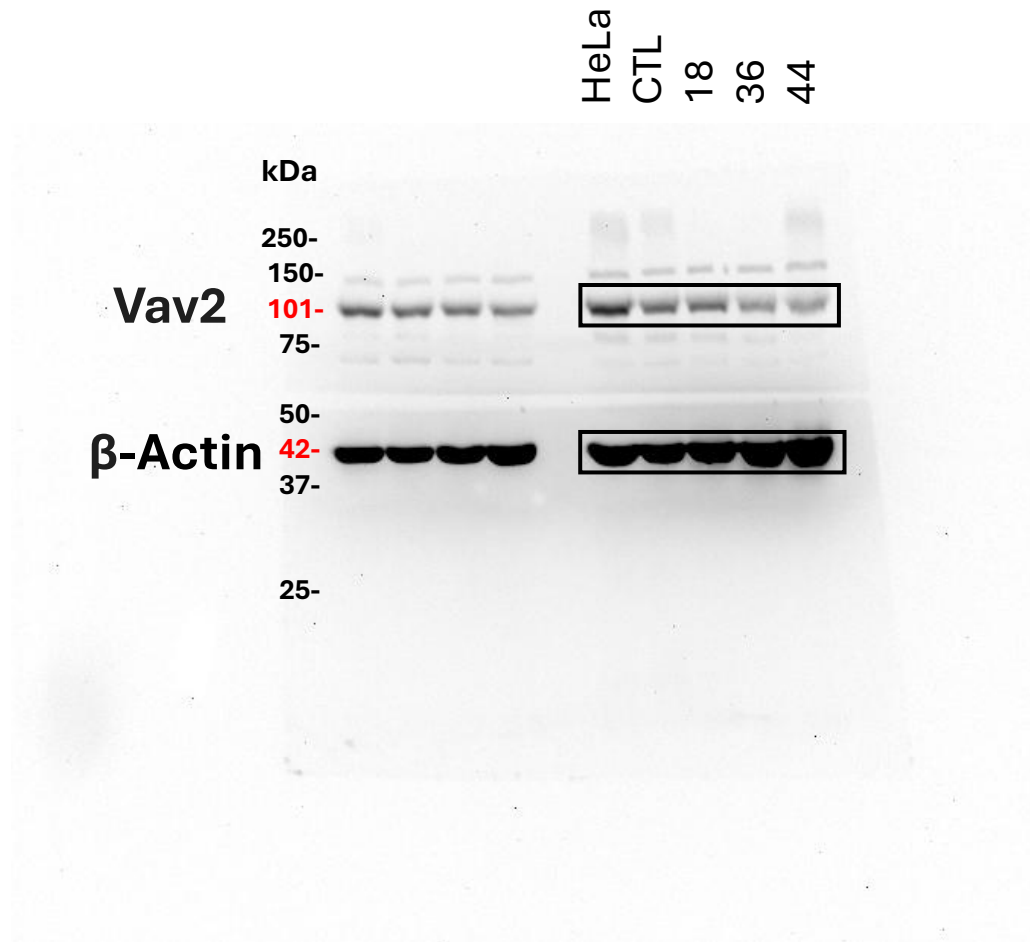

Figure 4C

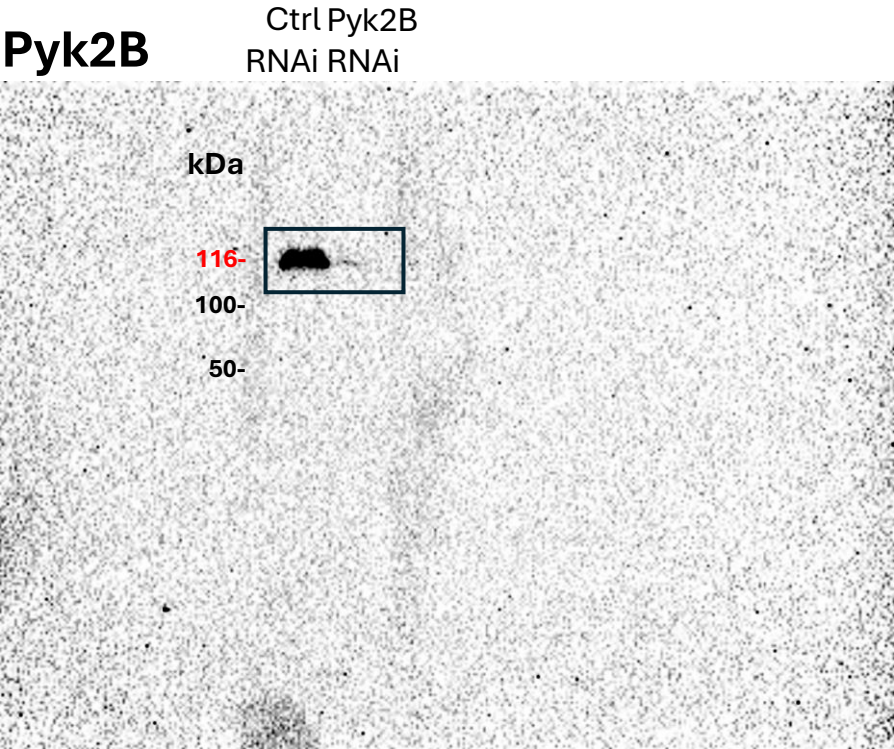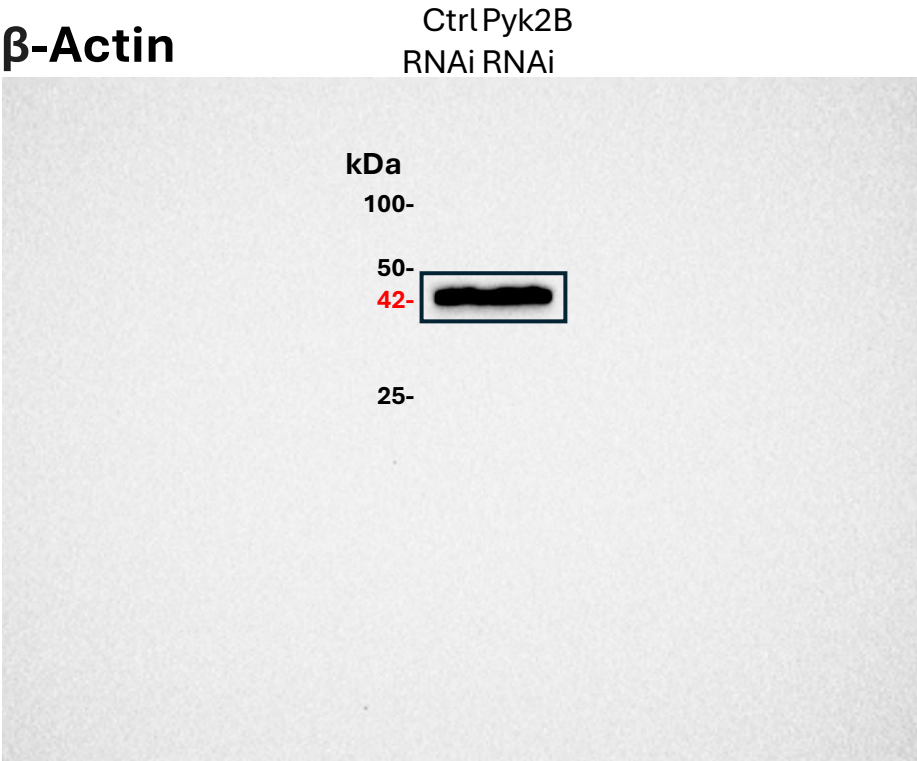

# Figure 5D

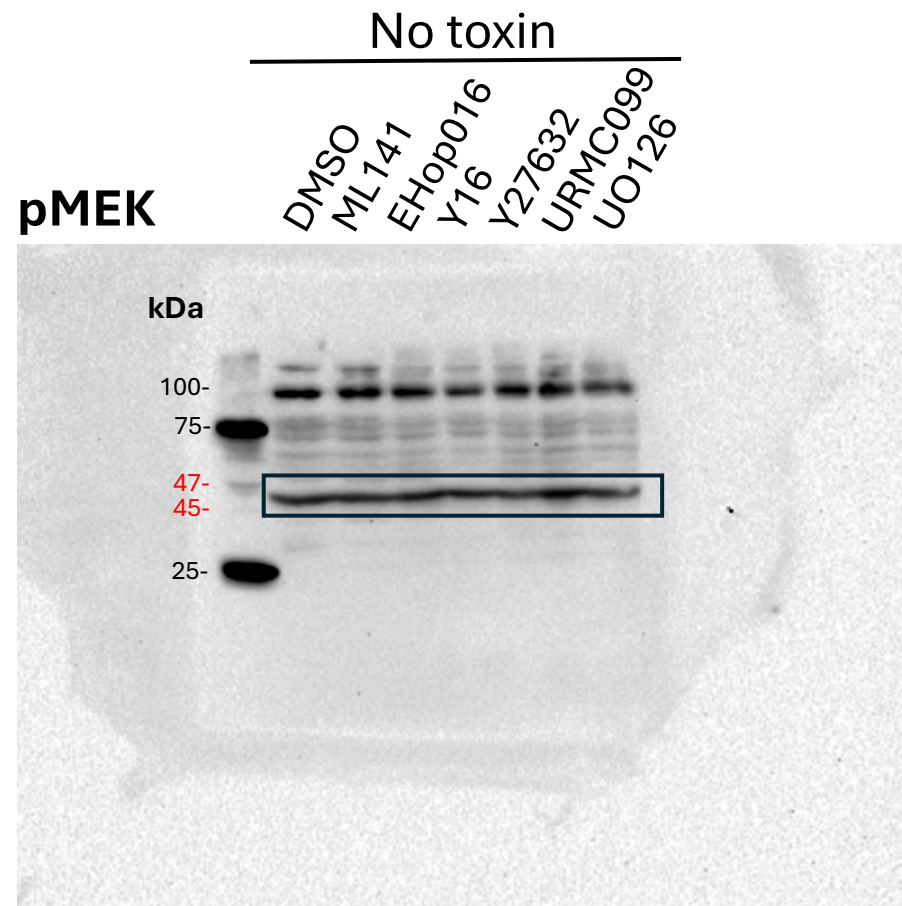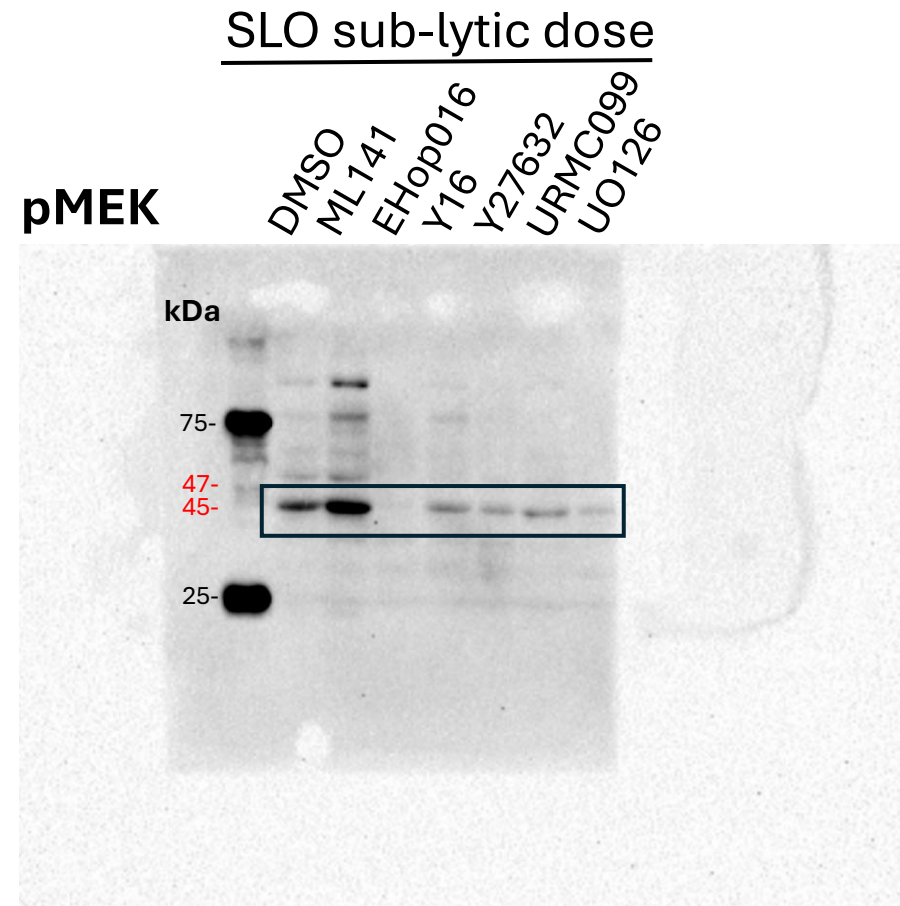

Figure 5D

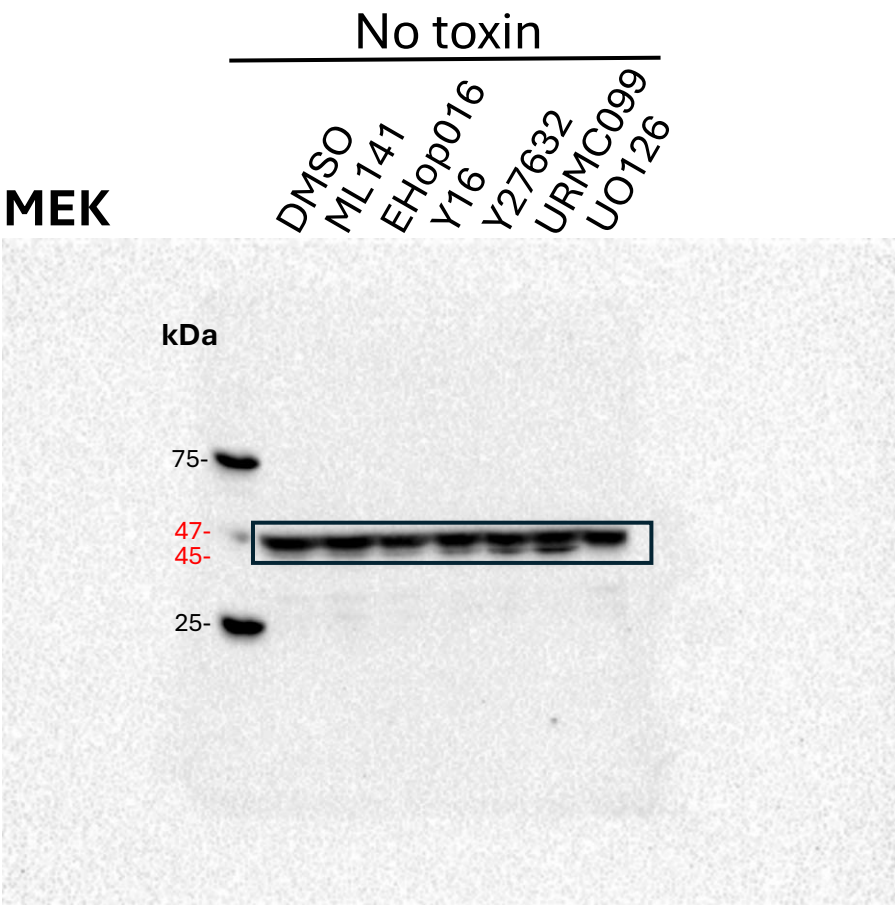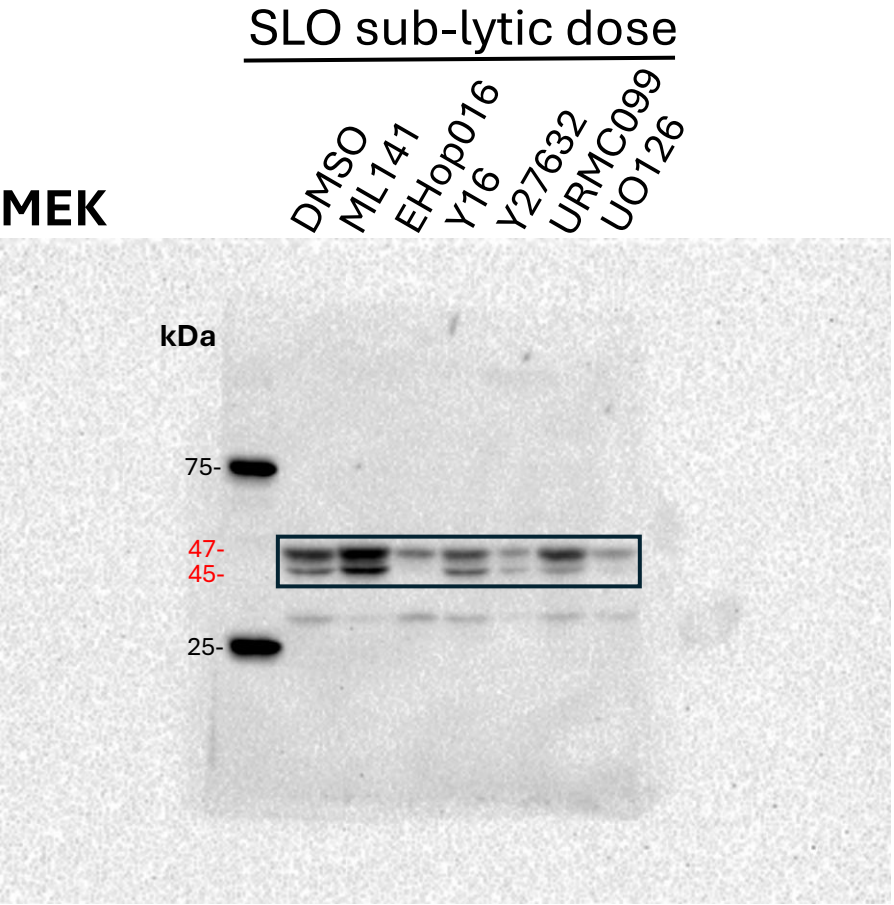

Figure 5D

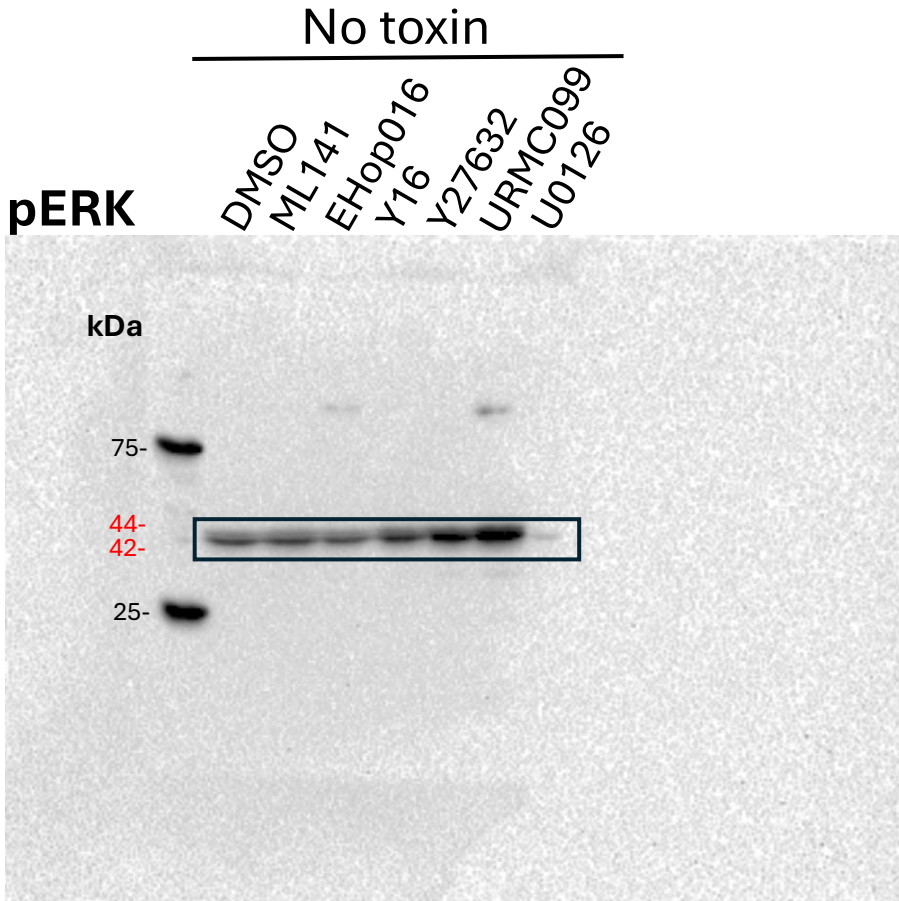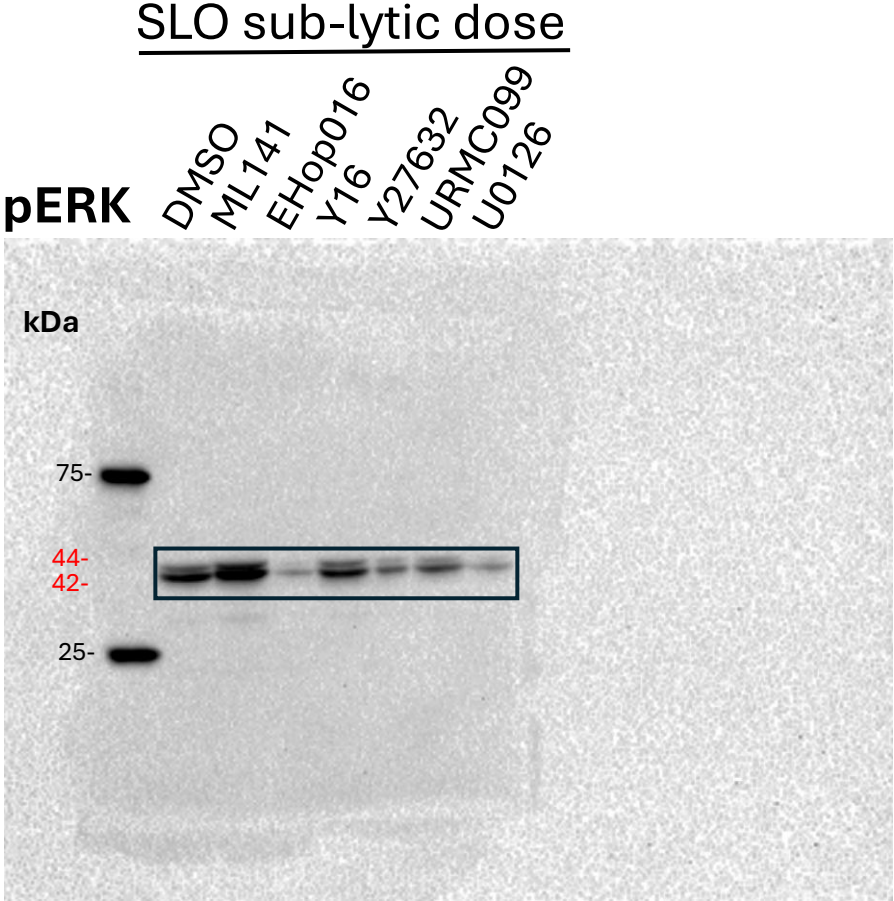

Figure 5D

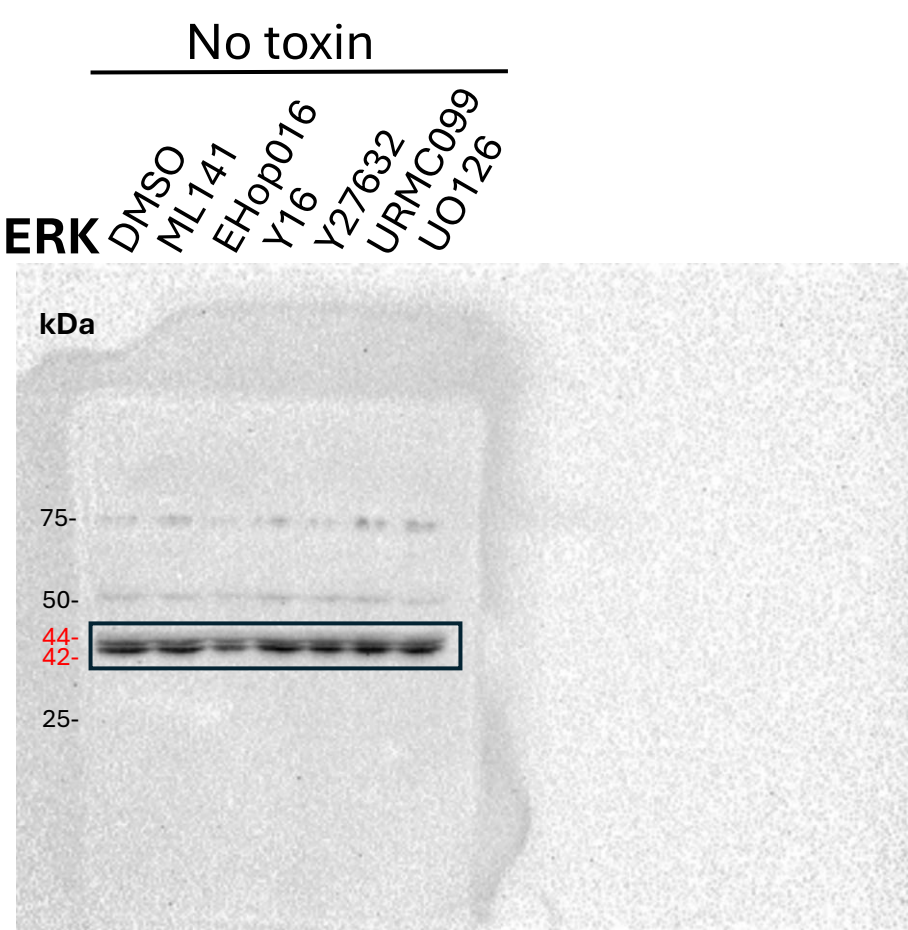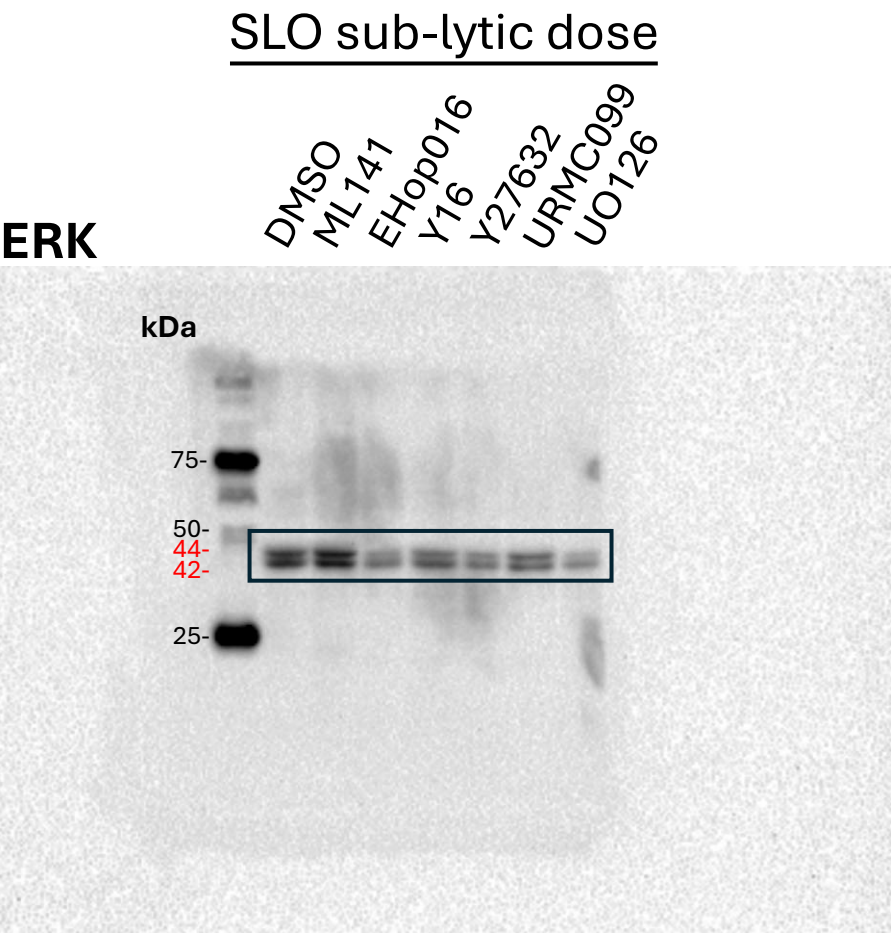

Figure 5D

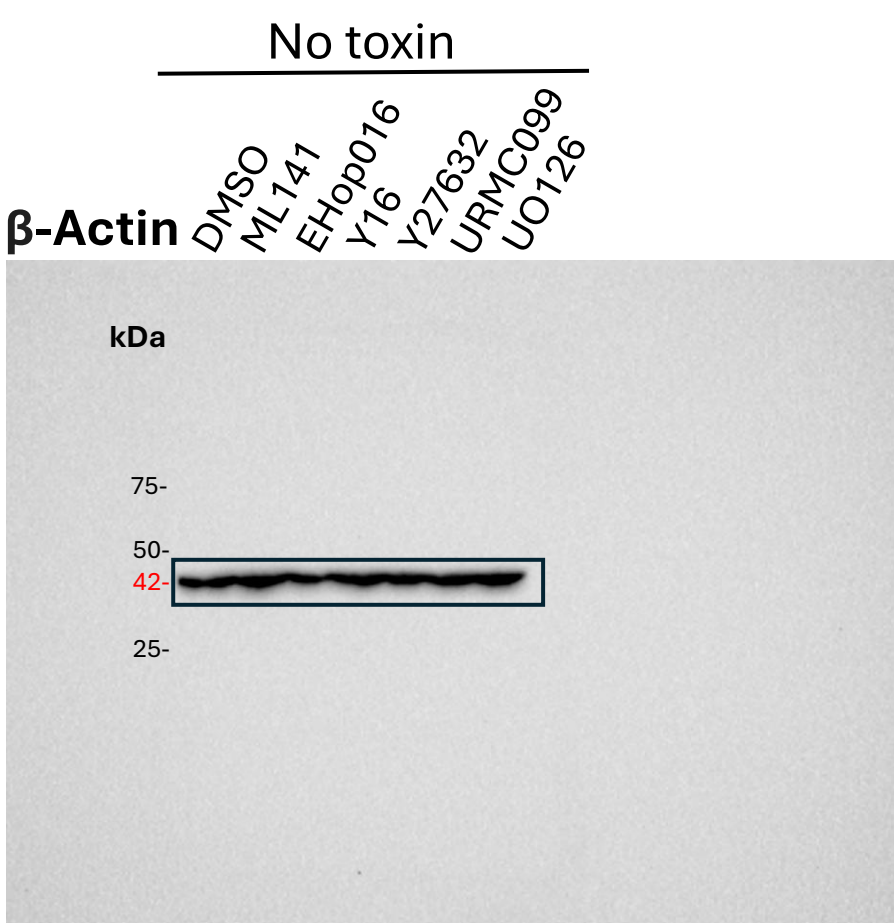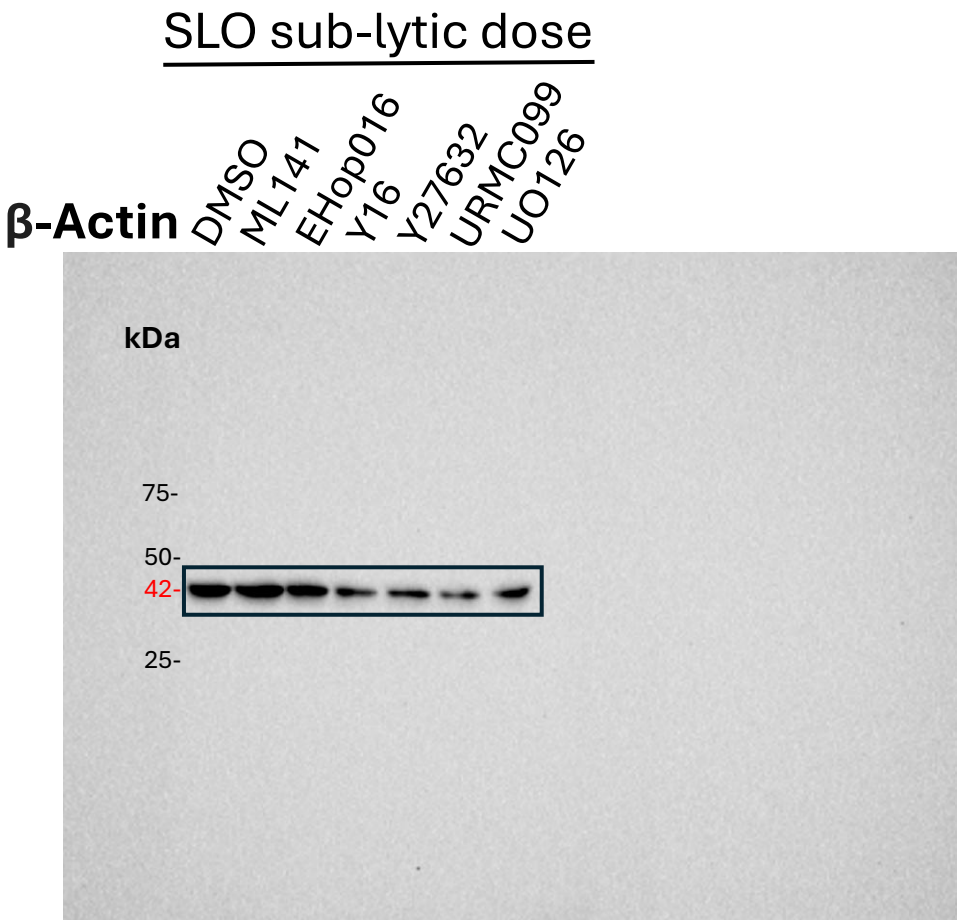

Figure 6A

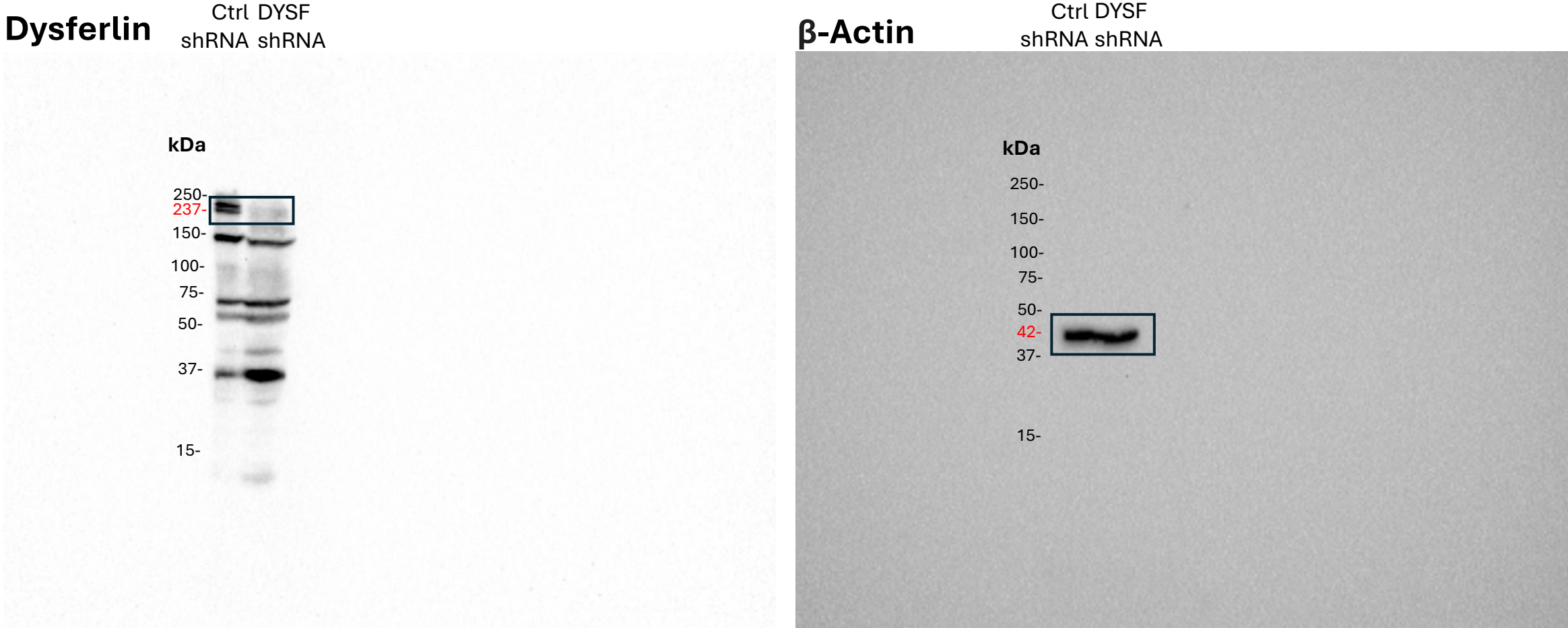

Figure 6C

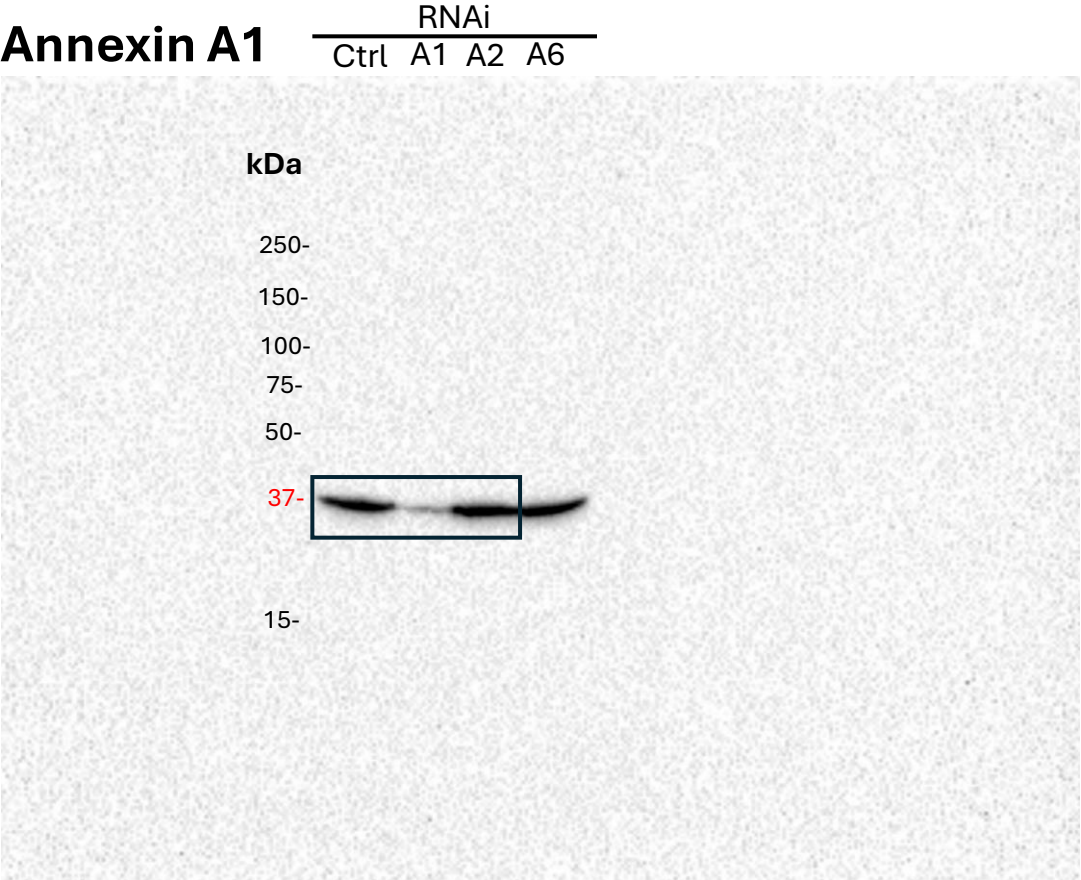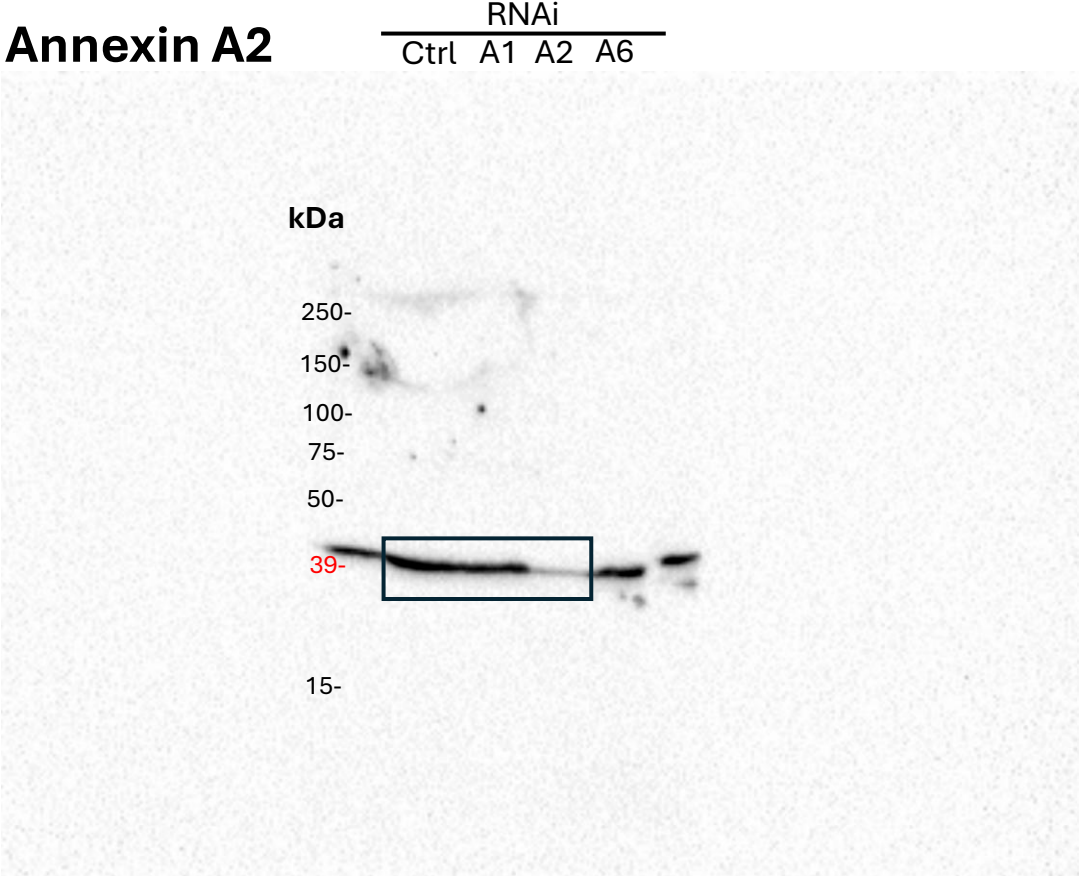

Figure 6C

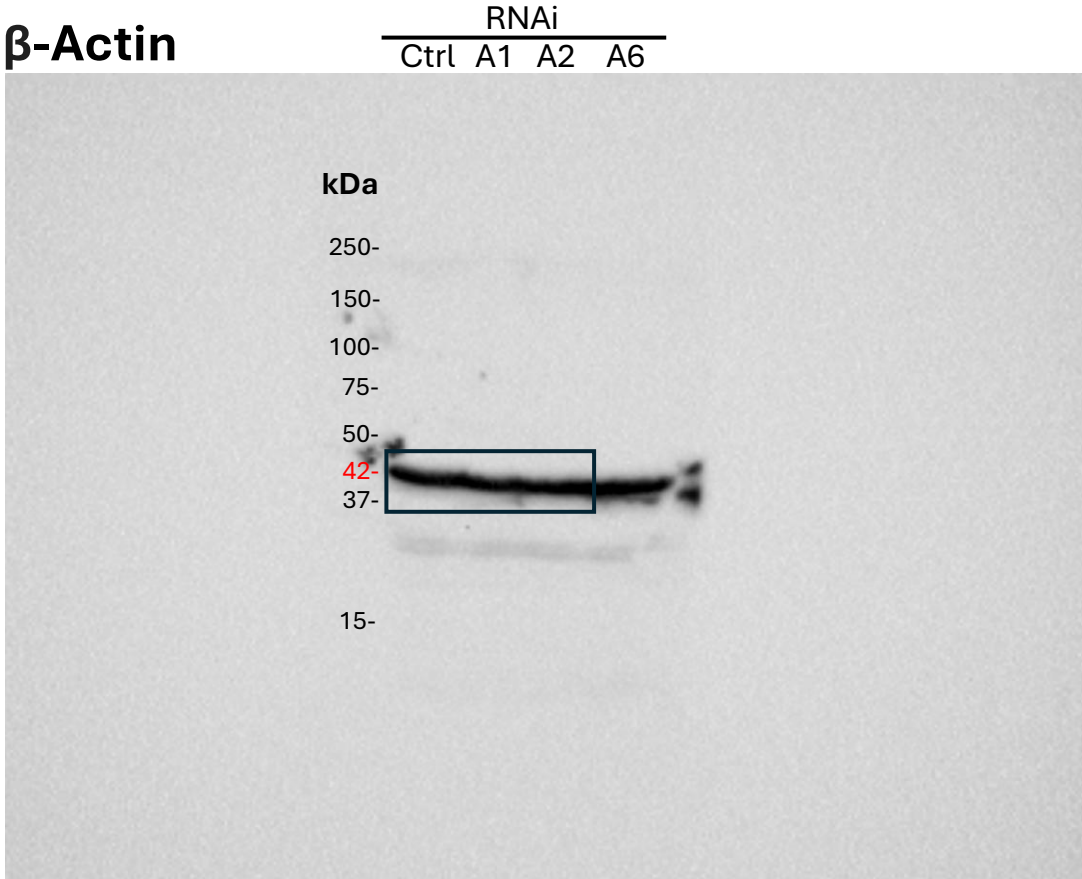

Supplement: Supplementary file 2 [file LSA_2026_03633_SdataF3_F4_F5_F6.pdf]
