# Supplementary material for: Vav2 is a master regulator of repair against bacterial pore-forming toxins
Source: Life Sci Alliance. 2026 Jul 9;9(9):e202603633. doi: 10.26508/lsa.202603633 (PMC13351264; doi:10.26508/lsa.202603633)
Supplement: Supplementary file 3 [file LSA-2026-03633_TableS2.docx]

**Supplementary Table S2. Levene’s test on data in Fig 3C.**

| **Assay #** | **F stat** | **p value** | **n control cells** | **n Vav2 cells** |
| --- | --- | --- | --- | --- |
| 1 | 0.0127 | 0.9107 | 64 | 74 |
| 2 | 0.2951 | 0.5885 | 51 | 75 |
| 3 | 0.7153 | 0.3998 | 103 | 66 |
