# Supplementary material for: Vav2 is a master regulator of repair against bacterial pore-forming toxins
Source: Life Sci Alliance. 2026 Jul 9;9(9):e202603633. doi: 10.26508/lsa.202603633 (PMC13351264; doi:10.26508/lsa.202603633)
Supplement: Reviewer comments [file LSA-2026-03633_review_history.pdf]

Vav2 is a master regulator of repair against bacterial pore-forming toxins.

Victor Kayejo, Ashlee Hensley, Tejal Katore, and Peter Keyel
DOI: https://doi.org/10.26508/lsa.202603633

Corresponding author(s): Peter Keyel, Texas Tech University

Review Timeline:

Submission Date:	2026-01-14
Editorial Decision:	2026-02-13
Revision Received:	2026-05-12
Editorial Decision:	2026-06-10
Revision Received:	2026-06-19
Accepted:	2026-06-24

Scientific Editor: Sarita Hebbar

Transaction Report:

(Note: With the exception of the correction of typographical or spelling errors that could be a source of ambiguity, letters and reports are not edited. The original formatting of letters and referee reports may not be reflected in this compilation.)

February 13, 2026

Re: Life Science Alliance manuscript #LSA-2026-03633-T

Dr. Peter Keyel
Texas Tech University
Biological Sciences
Unknown
Lubbock, TX 79409

Dear Dr. Keyel,

Thank you for submitting your manuscript entitled "Vav2 is a master regulator of repair against bacterial pore-forming toxins." to Life Science Alliance.

The manuscript was assessed by three expert reviewers, whose comments are appended to this letter. As you will read, the reviewers commented that most claims in your manuscript are supported by evidence, and positively highlight the study design and experiments. But Reviewers 2 and 3 have raised some concerns that preclude publication at this stage. Based on their overall comments, we invite you to submit a revised manuscript.

We agree that you must address the concerns of Reviewer 2 (figures 3C and S1) and 3 (figure 6) regarding figure presentation and analyses. Further we also would like to direct your attention to point 5 (Reviewer 2) on the generation of the VAv2 CRISPR line and concur that you must comment on the homogeneity of the KD in your manuscript.
We agree with the reviewers that you must moderate your claims specifically related to dysferlin-mediated membrane repair, and effect of MEK inhibitor (Reviewer 2, points 6, 7) and accurately describe Calcium dependent repair (Reviewer 3 and Reviewer 2, comment 4).
We concur with Reviewer 2 that use of CRISPR knockouts to support findings on CDC and involvement of other regulators (points 1, 2) would strengthen the manuscript.

When submitting the revision, please include a letter addressing the reviewers' comments point by point. While a rebuttal must respond to all points in some form, additional experiments to resolve these points, other than indicated above, are not required.

The typical timeframe for revisions is three months. Please note that papers are generally considered through only one revision cycle, so strong support from the referees on the revised version is needed for acceptance.

I would be happy to discuss the revision in more detail via email or phone/videoconferencing. Please let me know which option you prefer, if any.

To upload the revised version of your manuscript, please log in to your account: https://lsa.msubmit.net/cgi-bin/main.plex

You will be guided to complete the submission of your revised manuscript and to fill in all necessary information. Please get in touch in case you do not know or remember your login name.

The typical timeframe for revisions is three months. Please note that papers are generally considered through only one revision cycle, so strong support from the referees on the revised version is needed for acceptance.

While you are revising your manuscript, please also attend to the below editorial points to help expedite the publication of your manuscript. Please direct any editorial questions to the journal office. When submitting the revision, please include a letter addressing the reviewers' comments point by point.

Thank you for this interesting contribution to Life Science Alliance. We hope that the comments below will prove constructive as your work progresses, and we are looking forward to receiving your revised manuscript.

Sincerely,

Sarita Hebbar, PhD
Scientific Editor
Life Science Alliance
http://www.lsajournal.org

A. THESE ITEMS ARE REQUIRED FOR REVISIONS

-- A letter addressing the reviewers' comments point by point.

-- An editable version of the final text (.DOC or .DOCX) is needed for copyediting (no PDFs).

-- High-resolution figure, supplementary figure and video files uploaded as individual files: See our detailed guidelines for preparing your production-ready images, https://www.life-science-alliance.org/authors

-- Summary blurb (enter in submission system): A short text summarizing in a single sentence the study (max. 200 characters including spaces). This text is used in conjunction with the titles of papers, hence should be informative and complementary to the title and running title. It should describe the context and significance of the findings for a general readership; it should be written in the present tense and refer to the work in the third person. Author names should not be mentioned.

-- By submitting a revision, you attest that you are aware of our payment policies found here: https://www.life-science-alliance.org/copyright-license-fee

B. MANUSCRIPT ORGANIZATION AND FORMATTING:

Full guidelines are available on our Instructions for Authors page, https://www.life-science-alliance.org/authors

We encourage our authors to provide original source data, particularly uncropped/-processed electrophoretic blots and spreadsheets for the main figures of the manuscript. If you would like to add source data, we would welcome one PDF/Excel-file per figure for this information. These files will be linked as supplementary "Source Data" files.

IMPORTANT: It is Life Science Alliance policy that if requested, original data images must be made available. Failure to provide original images upon request will result in unavoidable delays in publication. Please ensure that you have access to all original microscopy and blot data images before submitting your revision.

Reviewer #1 (Comments to the Authors (Required)):

This paper reports that the Rac GEF Vav2 accounts for most of the calcium-dependent repair of membrane damage. Elimination of Vav2 sensitizes cells to toxin-dependent damage. Vav2 acts by modulating the MLK3-MEK membrane repair pathways. Overall, the results support the authors conclusions. I have no major issues.

Reviewer #2 (Comments to the Authors (Required)):

This study aims to examine the role of the Rac proteins in regulating the susceptibility of cells to various cytolytic toxins. Authors report that the Rac protein Vav2 is exclusively involved in regulating CDC susceptibility, which they report is mediated through MEK-dependent pathway for repairing toxin-injured cell membrane. This defect due to Vav2 inhibition can be rescued by MEK activation, but the deficit did not synergize with the knockdown of dysferlin and annexin pathways for cell membrane repair. The findings are of broad interest and studies are thorough in their design and the results are supported by the experimental findings. There are a few claims that need additional considerations that I have noted below.
1) Given the limited availability of highly selective small-molecule inhibitors of Vav2, additional experiments would help clarify whether the effects of EHop016 are specifically mediated through Vav2 inhibition. In particular, comparing CDC in a Vav2 CRISPR knockdown cell line with that observed in drug-treated cells would strengthen this conclusion. Presenting the data in Figure 3C as HU/mL, consistent with other figures, would also improve clarity. Additionally, treating the knockdown cells with the drug could determine whether CDC sensitivity is further reduced, which would help rule out potential Vav2-independent effects.
2) The proposed relationships between Vav2 and other regulators, including MEK, MLK3, dysferlin, and annexins, are currently based on drug-treatment data alone. Validation of these findings using CRISPR-deleted cells would strengthen the evidence supporting these interactions and help establish their specificity.
3) Given that cell repair occurs within minutes, whereas the assays used here assess cell survival (rather than repair) and are performed more than half an hour after injury, downstream events following an initial repair deficit may influence the observed outcomes. The authors should therefore acknowledge this as a limitation in extending the claims from this study to other studies that assess membrane repair acutely.
4) The manuscript states that Vav2 is activated by Ca^{2+}; however, the biochemical basis of this activation is not described. Providing supporting biochemical data, along with evidence of a Ca^{2+}-dependent change in Vav2 activation, is needed.
5) There are a few concerns regarding the Vav2 CRISPR cell line generation and characterization
a) Control cell line is showed low Vav2 protein expression as compared to parental cells.
b) Quantification across multiple replicates to support the claim of 67% KD is missing.

c) Stable KD of Vav2 should be demonstrated by quantification across 1-2 passages.
d) To establish that the partial Vav2 KD is not due to a mixed culture, genomic DNA sequencing or immunostaining to assess homogeneity of Vav2 KD is needed.
6) The conclusion that Vav2 regulates dysferlin-mediated membrane repair is not fully supported by the presented data, as inhibition of Vav2 appears to increase CDC sensitivity independently of dysferlin expression. Additional evidence would help clarify the relationship between Vav2 activity and dysferlin-dependent repair mechanisms.
7) In Figure 5B, the data obtained using the MEK3 inhibitor are based on only three samples and show substantial variability (greater than two-fold) between replicates. This level of variability limits the robustness of the conclusions drawn in Figure 4D regarding the relationship between Vav2 and MEK3, and additional replicates would strengthen this claim.
8) Figure S1A does not clearly demonstrate the reported disruption of F-actin following the various treatments. Providing more quantitative analysis or representative images would help support this interpretation.

Reviewer #3 (Comments to the Authors (Required)):

This manuscript provides compelling data to indicate that:
1. Rac1 inhibition sensitizes cells to the SLO and PFO cholesterol-dependent cytolysins (CDC's) The sensitization was not observed when Rho or Cdc42 were inhibited.
2. The role of Rac1 in repair is regulated by Vav2, a Rac GEF.
3. The role of Vav2 in repair appeared to be independent of tyrosine kinases known to phosphorylate Vav2. Instead, Vav2 appears to be activated by CDC-dependent Ca2+ influx.
4. Vav acts upstream of the MLK3-MEK signaling pathway.
5. The Vav-dependent repair pathway is independent or (or parallel to) the ceramide protection pathway.
6. Vav regulates the dysferlin-mediated repair pathway.

Overall, this is an interesting, significant, and well designed study with clear experiments, experimental results, and interpretations.
The only Figure that I struggled with was Figure6, and in particular, panels E and F.
The authors say that the Vav inhibition delays Annexin recruitment to the membrane, but I don't see this. Can the data be presented in a more objective way and ideally, quantified?
My other minor issue was with the text claiming that the Vav pathway was responsible for 90% of Ca2+-dependent repair. This assessment is not presented in a rigorous manner. Keep in mind that the LD50 curve fits are loose approximations since many of the lysis curves in the supplemental data are incomplete. This is not a big deal in my perspective as the effects in the raw supplemental data are clear. However, I think it is a reach to try to quantify the role of a pathway in a process, especially under a single set of specific cell culture conditions.

Minor comments:
Line 63. My understanding is that Cholesterol is considered central to the mechanism, but researchers increasingly view it as part of a broader lipid-dependent process rather than a simple one-to-one binding interaction.
Line 94. I think copies should be copines?
Line 140: Odd use of the word 'logistically'. I assume you mean that you modeled the dose response curves on a logarithmic scale.

Reviewer #2 (Comments to the Authors (Required)):

1) Given the limited availability of highly selective small-molecule inhibitors of Vav2, additional experiments would help clarify whether the effects of EHop016 are specifically mediated through Vav2 inhibition. In particular, comparing CDC in a Vav2 CRISPR knockdown cell line with that observed in drug-treated cells would strengthen this conclusion. Presenting the data in Figure 3C as HU/mL, consistent with other figures, would also improve clarity. Additionally, treating the knockdown cells with the drug could determine whether CDC sensitivity is further reduced, which would help rule out potential Vav2-independent effects.

We added a panel to Fig 3C (now Fig 3D) showing the results in HU/mL. To the request we combine the inhibitor with the CRISPR cells, we note that our knockdown was about 67% effective, suggesting that one of the three Vav2 alleles was maintained. In our opinion, combining the inhibitor with these cells would not provide additional information because it would be unclear if there were Vav2-independent effects, or if the inhibitor was targeting the residual Vav2 within the cell. Since the inhibitor can transiently block all the Vav in the cell (vs 67% from the CRISPR cells), and has greater translational capacity, we think this tool is the best one for subsequent experiments. Given the sensitivity of cells to two inhibitors that target Vav2, no sensitivity for Rac inhibitors that fail to target Vav2, and combined with our CRISPR cells, we think it is reasonable to conclude the inhibitor acts via Vav2 inhibition. We have added the partial CRISPR knockdown as a limitation in the discussion.

2) The proposed relationships between Vav2 and other regulators, including MEK, MLK3, dysferlin, and annexins, are currently based on drug-treatment data alone. Validation of these findings using CRISPR-deleted cells would strengthen the evidence supporting these interactions and help establish their specificity.

Please see point 1 above for our choice to use the Vav2 inhibitor instead of the Vav2 CRISPR cells. For the other inhibitors, we previously validated the MEK and MLK3 inhibitors against siRNA in Ray et al 2022. We expect deletion of MEK1 and MEK2 by CRISPR to be lethal.

3) Given that cell repair occurs within minutes, whereas the assays used here assess cell survival (rather than repair) and are performed more than half an hour after injury, downstream events following an initial repair deficit may influence the observed outcomes. The authors should therefore acknowledge this as a limitation in extending the claims from this study to other studies that assess membrane repair acutely.

We disagree with the reviewer that this is a limitation because of our previously published results. We tested the time aspect in Ray et al 2018. We found no change in killing for SLO between 5 and 30 min. Prior to 5 min, we observe significant intra-assay variability, which we attribute to toxin diffusion/cell binding. Since this issue will impact others' use of toxins in repair, too, we believe our claims can be extended to other studies using toxins. When it comes to other mechanisms of causing damage, we think the type of damage is the major source of differences between our claims and those of others. We have clarified in the manuscript that we are focused on repair to CDCs, instead of repair to everything.

We further note that we had the same findings here for PFO and SLO. In contrast to SLO, PFO takes 30 min to achieve maximal killing. That we did not observe a difference between these two toxins suggests events occurring 5-30 min later are not a meaningful source of variability in assays. Finally, our assay also detects transiently permeabilized cells (see Keyel et al, PMID: 21693578). These are not reported because they generally mirror findings with the dead cells (eg we observe a peak in transiently permeabilized cells at concentrations 2-4 fold below cell death, which decreases as cells die) and because we anticipate that adding (and discussing) these data would add confusion and mental load on the reader without a meaningful addition to the story.

4) The manuscript states that Vav2 is activated by Ca^{2+}; however, the biochemical basis of this activation is not described. Providing supporting biochemical data, along with evidence of a Ca^{2+}-dependent change in Vav2 activation, is needed.

We stated (line 220-221) that Vav is activated “after Ca^{2+} influx” for Ca^{2+}-dependent repair, not activated “by Ca^{2+} influx”. We do not make any claims that Vav2 is directly activated by Ca^{2+}, and suggest that identifying the connection remains an important and outstanding question. While it is possible that Ca^{2+}-influx activates a tyrosine kinase that activates Vav, there could be other membrane perturbations that trigger tyrosine phosphorylation or some other activation mechanism of Vav. Support for our assertion that Vav is activated after Ca^{2+} flux comes from our data showing the lack of additive effects between EGTA and Vav. We interpret these data to indicate Vav acts in the Ca^{2+}-dependent repair pathways. Many of these other pathways we investigated (eg MEK, annexins) were previously established to be Ca^{2+}-dependent.

We rephrased line 220-221 to read ‘Vav-dependent repair is activated after Ca^{2+} influx’ instead of Vav.

5) There are a few concerns regarding the Vav2 CRISPR cell line generation and characterization
a) Control cell line is showed low Vav2 protein expression as compared to parental cells.

We agree that the CRISPR control line showed less Vav2 protein expression compared to the parental cells. To control for this, we used these CRISPR control cells for determining the amount of Vav2 remaining in the cells, for normalizing our data, and for interpreting our killing assays. Based on our killing data, the difference in expression between the parental line and the CRISPR controls was not biologically relevant in this system.

b) Quantification across multiple replicates to support the claim of 67% KD is missing.

We added the quantification of our independent replicates in Fig 3B. Each replicate was from a different passage. We further thawed the cells and performed an additional replicate for the control and CRISPR clone 44. We also note that the variability in the CRISPR clone 44 did NOT vary with time (eg the lowest Vav2 expression was NOT the first passage).

c) Stable KD of Vav2 should be demonstrated by quantification across 1-2 passages.

Please see 5b, above.

d) To establish that the partial Vav2 KD is not due to a mixed culture, genomic DNA sequencing or immunostaining to assess homogeneity of Vav2 KD is needed.

We performed immunostaining of the Vav2 knockdown cells and the CRISPR control, which is added as Fig 3C. We observed even staining across the cells, suggesting that the knockdown was not due to a mixed culture. We further tested if the variance in Vav2 expression was different than the variance in control cells using Levene's test. We found no difference in variance, which suggests any fluctuations in Vav2 levels in these cells is the expected amount of variance, and not a mixed culture.

6) The conclusion that Vav2 regulates dysferlin-mediated membrane repair is not fully supported by the presented data, as inhibition of Vav2 appears to increase CDC sensitivity independently of dysferlin expression. Additional evidence would help clarify the relationship between Vav2 activity and dysferlin-dependent repair mechanisms.

We have moderated these statements. We interpret the lack of additive effects when both Vav2 and dysferlin are absent to indicate they are in the same pathway.

7) In Figure 5B, the data obtained using the MEK3 inhibitor are based on only three samples and show substantial variability (greater than two-fold) between replicates. This level of variability limits the robustness of the conclusions drawn in Figure 4D regarding the relationship between Vav2 and MEK3, and additional replicates would strengthen this claim.

We acknowledge the variability in Fig 5B and Fig 5C, and have added this as a limitation (line 339). We note additional strength for the claim also lies in the finding that SLO and PFO phenocopy each other in these two panels, and the ~2-3 fold change with U0126 and URMC099 is consistent with our prior published results (Ray et al, 2022).

8) Figure S1A does not clearly demonstrate the reported disruption of F-actin following the various treatments. Providing more quantitative analysis or representative images would help support this interpretation.

We added quantitation to Fig S1A, reported now as Fig S1B.

Reviewer #3 (Comments to the Authors (Required)):

The only Figure that I struggled with was Figure6, and in particular, panels E and F.
The authors say that the Vav inhibition delays Annexin recruitment to the membrane, but I don't see this. Can the data be presented in a more objective way and ideally, quantified?

We quantitated these data and added that to Fig 6.

My other minor issue was with the text claiming that the Vav pathway was responsible for 90% of Ca2+-dependent repair. This assessment is not presented in a rigorous manner. Keep in mind

that the LD50 curve fits are loose approximations since many of the lysis curves in the supplemental data are incomplete. This is not a big deal in my perspective as the effects in the raw supplemental data are clear. However, I think it is a reach to try to quantify the role of a pathway in a process, especially under a single set of specific cell culture conditions.

We arrived at the 90% figure by comparing the LC50 with Ehop to that of EGTA and untreated cells. We made this statement more qualitative by saying “almost all”. Despite the lack of statistical significance between EGTA and Ehop, we chose “almost all” instead of “all” to temper the statement, and align with our earlier calculation.

Minor comments:
Line 63. My understanding is that Cholesterol is considered central to the mechanism, but researchers increasingly view it as part of a broader lipid-dependent process rather than a simple one-to-one binding interaction.

We agree that CDC binding is more complex than described in our introduction. While neighboring lipids (and carbohydrates) in cells can alter membrane binding, oligomerization, and pore-formation, cholesterol remains central to SLO and PFO binding. Since our manuscript does not focus on CDC binding, we kept it high-level in our introduction to avoid confusion.

Line 94. I think copies should be copines?
We thank the reviewer for catching this typo.

Line 140: Odd use of the word 'logistically'. I assume you mean that you modeled the dose response curves on a logarithmic scale.

We are using a logistic model, which models the S-shaped curve. We rephrased to “we fit dose-response curves to a logistic model” for clarity.

June 10, 2026

RE: Life Science Alliance Manuscript #LSA-2026-03633-TR

Dr. Peter Keyel
Texas Tech University
Biological Sciences
Unknown
Lubbock, TX 79409

Dear Dr. Keyel,

Thank you for submitting your revised manuscript entitled "Vav2 is a master regulator of repair against bacterial pore-forming toxins.". Your manuscript was evaluated by two of the original reviewers whose comments are appended below. As you will read, both have acknowledged your revised manuscript. However Reviewer 2 noted some insufficiently addressed concerns that you must resolve with appropriate edits in your manuscript text. We do not expect any further experiments.

Overall, we would be happy to publish your paper in Life Science Alliance pending resolution of the above minor points and final revisions necessary to meet our formatting guidelines. We request you to submit a revised manuscript document with changes highlighted along with a point-by-point response to the reviewer's comments.

MANUSCRIPT ORGANIZATION AND FORMATTING:

To avoid unnecessary delays in the acceptance and publication of your paper, please read the following information carefully. Full guidelines are available on our Instructions for Authors page, https://www.life-science-alliance.org/authors

Submission of a paper that does not conform to Life Science Alliance guidelines will delay the acceptance of your manuscript.

-Please integrate the ethical approval statement into the existing information on page 19, line 391 onwards, and delete the statement repeated on page 25, line 525.
-Please elaborate on methods for Actin quantification.
-Please provide sequence for negative control guide RNA and guide RNA targeting Vav2 in the methods section.
-Please add the X and Bluesky handles of your host institute/organisation, as well as your own, and/or one of the authors, in our system
-Please rename "COMPETING INTERESTS" to "Conflict of Interest" and "AVAILABILITY OF DATA AND MATERIALS" to "Data Availability."
-Please be sure that the authorship listing and order is correct.

We encourage our authors to provide original source data, particularly uncropped/-processed electrophoretic blots and spreadsheets for the main figures of the manuscript. If you would like to add source data, we would welcome one PDF/Excel-file per figure for this information. These files will be linked as supplementary "Source Data" files.

It is Life Science Alliance policy that if requested, original data images must be made available to the editors. Failure to provide original images upon request will result in unavoidable delays in publication. Please ensure that you have access to all original data images prior to final submission.

If you are planning a press release on your work, please inform us immediately to allow informing our production team and scheduling a release date.

We welcome submissions of potential cover images for the issue of LSA in which your work would appear. If you have high quality images associated with this work, please feel free to email these, with a caption, to the journal office.

LSA encourages authors to provide a 30-60 second video where the study is briefly explained. These videos will be appear embedded with the manuscript online at Life Science Alliance, and on social media to promote the published paper and authors (for examples, see https://docs.google.com/document/d/1-UWCfbE4pGcDdcgzcmiuJl2XMBJnxKYeqRvLLrLSo8s/edit?usp=sharing). Corresponding or first-authors are welcome to submit the video. Please submit only one video per manuscript. The video can be emailed to contact@life-science-alliance.org

FINAL FILES:

To upload the final version of your manuscript, please log in to your account: https://lsa.msubmit.net/cgi-bin/main.plex
You will be guided to complete the submission of your revised manuscript and to fill in all necessary information. Please get in touch in case you do not know or remember your login name.

The following items are required for acceptance.

-- An editable version of the final text (.DOC or .DOCX) is needed for copyediting (no PDFs).

-- High-resolution figure, supplementary figure and video files uploaded as individual files: See our detailed guidelines for preparing your production-ready images, https://www.life-science-alliance.org/authors

-- Summary blurb (enter in submission system): A short text summarizing in a single sentence the study (max. 200 characters including spaces). This text is used in conjunction with the titles of papers, hence should be informative and complementary to the title. It should describe the context and significance of the findings for a general readership; it should be written in the present tense and refer to the work in the third person. Author names should not be mentioned.

The license to publish form must be signed before your manuscript can be sent to production. A link to the license to publish form will be available to the corresponding author only. Please take a moment to check your funder requirements.

Reviews, decision letters, and point-by-point responses associated with peer-review at Life Science Alliance will be published, alongside the manuscript. If you do want to opt out of having the reviewer reports and your point-by-point responses displayed, please let us know immediately.

Thank you for your attention to these final processing requirements. Please revise and format the manuscript and upload materials as soon as you are able.

Thank you for this interesting contribution to the literature. We look forward to publishing your paper in Life Science Alliance.

Sincerely,

Sarita Hebbar, PhD
Scientific Editor
Life Science Alliance
http://www.lsajournal.org

Reviewer #2 (Comments to the Authors (Required)):

The authors have made revisions that improve the manuscript; however, several of the prior concerns remain insufficiently addressed and require further clarification or revision, as outlined below.
1. The interpretation of the cell survival assay requires more careful framing. The assay employed (independent of the nature of initial membrane injury) measures overall cell survival, which is influenced not only by plasma membrane repair but also by downstream pathways activated following incomplete or failed repair that ultimately leads to cell death. Therefore, unless direct evidence demonstrating that the readout is exclusively determined by membrane repair processes (and not by downstream death pathways) is available, the conclusions should be appropriately qualified to reflect that the assay reports cell survival rather than membrane repair per se.
2. The interpretation of Vav2-related effects is limited by assay saturation. Because Vav2 inhibition drives the assay to maximal sensitivity (dotted line in many of the plots, which should be included in all plots), it prevents detection of any further increases in cell lysis, thereby constraining the ability to assess potential additive or synergistic effects of other repair pathways. This includes pathways mediated by calcium signaling, dysferlin, or annexins. As such, the current experimental design is not well suited to assess additive or non-additive pathways in the context of reduced repair capacity. To support such claims, gain-of-function experiments (as shown for the ceramide experiments) should be considered or definitive statements regarding the nature of interaction with these pathways should be tempered.
3. The conclusions regarding Vav2 knockdown and LC50 are not adequately supported by the presented CRISPR data. None of the CRISPR clones show a statistically significant reduction in Vav2 expression, while the CTL shows an ~300% reduction relative to HeLa cells. The stated conclusions are further undermined by the observed lack of concordance between Vav2 expression and LC50 across clones: clone 36 (~50% reduction relative to CTL) shows no change in LC50, whereas clone 44 (~67% reduction relative to CTL) shows a reduced LC50. Thus, the possibility that clone-specific effects, rather than Vav2 expression level per se, may be driving the observed phenotype cannot be ruled out, and the suitability of claims based on clone 44 should be reconsidered/validated e.g. by re-expressing Vav2.
4. For the data presented in Figure 3C, the analysis of Vav2 variance in control versus CRISPR knockout cell lines is referenced but not shown. These data should be presented to allow its evaluation by the readers.
5. The interpretation of F-actin measurements remains unclear. As previously noted, Figure S1A does not demonstrate a

reduction in phalloidin staining, and the additional data in Figure S1B are consistent with no change in total F-actin content. A detailed description of how total versus mean F-actin intensity was quantified, including image analysis methodology and normalization procedures, should be included. Importantly, the rationale for the apparent discrepancy between total and mean intensity measurements should be clarified.

6. For experiments in which outcome variability limits the reliability of small sample sizes (n=3), the results should be regarded as preliminary. For drawing definitive conclusions, the study design should be strengthened through appropriate power calculations to determine an adequate sample size and by incorporating additional data to achieve robust, conclusive findings, or by noting the preliminary nature of these findings

Reviewer #3 (Comments to the Authors (Required)):

The authors have fully addressed my previous points of concern.

Reviewer #2 (Comments to the Authors (Required)):

The authors have made revisions that improve the manuscript; however, several of the prior concerns remain insufficiently addressed and require further clarification or revision, as outlined below.

We thank the reviewer for the feedback to help strengthen our manuscript, and the time spent considering it.

1. The interpretation of the cell survival assay requires more careful framing. The assay employed (independent of the nature of initial membrane injury) measures overall cell survival, which is influenced not only by plasma membrane repair but also by downstream pathways activated following incomplete or failed repair that ultimately leads to cell death. Therefore, unless direct evidence demonstrating that the readout is exclusively determined by membrane repair processes (and not by downstream death pathways) is available, the conclusions should be appropriately qualified to reflect that the assay reports cell survival rather than membrane repair per se.

We have updated the methods (lines 468-471) to clarify that our assay measures cell membrane integrity over 30 min, which, based on our extensive prior validation of this technique, is a robust membrane repair assay. We further added our "PI low" data for Supplemental Fig S2 to demonstrate the repair aspects of our assay (discussed on lines 176-178).

We disagree with the reviewer that PI uptake is fundamentally a cell survival assay. It is fundamentally a membrane integrity assay. In our system, it does report cell survival, but to draw that conclusion, we validated the assay against mitochondrial function (eg MTT assay). In some cases, cells can die without loss of membrane integrity (eg Ninj1 knockout macrophages). Our assay would not detect these cells. In other cases, cells can survive membrane permeabilization (see new panels in Fig S2). Thus, we consider it incorrect to characterize it as a "cell survival assay".

We further assert that the process of membrane repair must encompass countering any proximal (eg <1 hour) cell death pathways that disrupt membrane integrity. Cellular mechanisms to rupture membrane integrity in this time span must overcome membrane repair and/or inactivate the signaling pathways, like the ones we describe in this manuscript. Furthermore, cell death signaling and execution pathways are not straightforward at the short time points used in our assays (eg the discovery of Ninj1-mediated membrane rupture, PANoptosis). These pathways all interact, and it is our position that treating them as separate pathways misses the underlying physiology. Absent the use of knockouts or neutralizing antibodies, no microscopic technique or other method can distinguish between the immediate activation of an endogenous PFT by an external PFT, vs membrane rupture by the external PFT (eg Ninj1 is activated by SLO, see Fig 3a in PMID: 33472215). We further note some repair pathways, like patch repair, are arguably "downstream cellular pathways", despite their contribution of membrane to resealing. If there is a signaling mechanism that disables a

programmed cell death pathway, our opinion is that is also part of membrane repair, because it is necessary to maintain membrane integrity (and survival), and it may occur faster than vesicle trafficking events. For these reasons we believe a dichotomy between "membrane repair processes" and "downstream death pathways" is neither a helpful nor practical distinction at proximal time points to membrane damage.

2. The interpretation of Vav2-related effects is limited by assay saturation. Because Vav2 inhibition drives the assay to maximal sensitivity (dotted line in many of the plots, which should be included in all plots), it prevents detection of any further increases in cell lysis, thereby constraining the ability to assess potential additive or synergistic effects of other repair pathways. This includes pathways mediated by calcium signaling, dysferlin, or annexins. As such, the current experimental design is not well suited to assess additive or non-additive pathways in the context of reduced repair capacity. To support such claims, gain-of-function experiments (as shown for the ceramide experiments) should be considered or definitive statements regarding the nature of interaction with these pathways should be tempered.

We added a statement about gain of function in our limitations paragraph (line 342), and clarified that our conclusions about additive effects are based on the killing curves (lines 274-276) that underlie the LC50 measurements (provided in the supplemental material). While we are unable to calculate the LC50 when the lowest toxin dose remains above 50% lysis, the curves themselves decline from maximal lysis at the lowest toxin doses. This allows comparison of the curves to assess additive or non-additive effects.

3. The conclusions regarding Vav2 knockdown and LC50 are not adequately supported by the presented CRISPR data. None of the CRISPR clones show a statistically significant reduction in Vav2 expression, while the CTL shows an ~300% reduction relative to HeLa cells. The stated conclusions are further undermined by the observed lack of concordance between Vav2 expression and LC50 across clones: clone 36 (~50% reduction relative to CTL) shows no change in LC50, whereas clone 44 (~67% reduction relative to CTL) shows a reduced LC50. Thus, the possibility that clone-specific effects, rather than Vav2 expression level per se, may be driving the observed phenotype cannot be ruled out, and the suitability of claims based on clone 44 should be reconsidered/validated e.g. by re-expressing Vav2.

We added a statement about the potential for clone-specific effects in our limitations paragraph (line 342). We disagree with the reviewer that our conclusions are "undermined by the observed lack of concordance between Vav2 expression and LC50 across clones". First, we note that clone 36 shows a trend to lower LC50 compared to CTL. So one possibility is that with a higher n, we would observe a statistical difference between clone 36 and control. Another possibility is that Vav2 exerts a threshold effect, such that once a minimum amount of Vav2 is present in the cell, it will show wild type or close to wild type behavior. Both of these possibilities are consistent with our conclusions. Since the results with the CRISPR clones further concords with our use of multiple Vav2 inhibitors, we think clone-specific effects are unlikely.

We did not perform post hoc statistical tests comparing to the control because it was normalized to 1 for each experiment.

4. For the data presented in Figure 3C, the analysis of Vav2 variance in control versus CRISPR knockout cell lines is referenced but not shown. These data should be presented to allow its evaluation by the readers.

We added the results of the Levene's tests for each of our biological replicates as Supplemental Table S2.

5. The interpretation of F-actin measurements remains unclear. As previously noted, Figure S1A does not demonstrate a reduction in phalloidin staining, and the additional data in Figure S1B are consistent with no change in total F-actin content. A detailed description of how total versus mean F-actin intensity was quantified, including image analysis methodology and normalization procedures, should be included. Importantly, the rationale for the apparent discrepancy between total and mean intensity measurements should be clarified.

We have updated this description in the methods (lines 513-518).

6. For experiments in which outcome variability limits the reliability of small sample sizes (n=3), the results should be regarded as preliminary. For drawing definitive conclusions, the study design should be strengthened through appropriate power calculations to determine an adequate sample size and by incorporating additional data to achieve robust, conclusive findings, or by noting the preliminary nature of these findings

To address concerns about variability limiting conclusions, we refer to some data differences as showing trends when there is no statistical significance. When our statistical analyses support the differences between groups, these samples have sufficient sample size to draw conclusions. For differences between groups, we used statistical analyses with the appropriate modifications for multiple comparisons and sample size, and displayed independent experiments. When there is large intra-assay variation, we typically lose statistical significance (eg clone 36 killing not being statistically different than CTL) instead of creating significance where there is none.

Reviewer #3 (Comments to the Authors (Required)):

The authors have fully addressed my previous points of concern.

We thank the reviewer for the feedback that helped strengthen our manuscript, and the time spent considering it.

June 24, 2026

RE: Life Science Alliance Manuscript #LSA-2026-03633-TRR

Dr. Peter Keyel
Texas Tech University
Biological Sciences
2901 Main St
Lubbock, TX 79409

Dear Dr. Keyel,

Thank you for submitting your Research Article entitled "Vav2 is a master regulator of repair against bacterial pore-forming toxins.". It is a pleasure to let you know that your manuscript is now accepted for publication in Life Science Alliance. Congratulations on this interesting work.

The final published version of your manuscript will be deposited by us to PubMed Central upon publication.

Your manuscript will now progress through copyediting and proofing. It is journal policy that authors provide original data upon request.

Your article will publish open access upon publication under a CC-BY license.

Reviews, decision letters, and point-by-point responses associated with peer-review at Life Science Alliance will be published, alongside the manuscript. If you do want to opt out of having the reviewer reports and your point-by-point responses displayed, please let us know immediately.

IMPORTANT: If you will be unreachable at any time, please provide us with the email address of an alternate author. Failure to respond to routine queries may lead to unavoidable delays in publication.

Scheduling details will be available from our production department. You will receive proofs shortly before the publication date. Only essential corrections can be made at the proof stage so if there are any minor final changes you wish to make to the manuscript, please let the journal office know now.

DISTRIBUTION OF MATERIALS:
Authors are required to distribute freely any materials used in experiments published in Life Science Alliance. Authors are encouraged to deposit materials used in their studies to the appropriate repositories for distribution to researchers.

You can contact the journal office with any questions, contact@life-science-alliance.org

Again, congratulations on a very nice paper. I hope you found the review process to be constructive and are pleased with how the manuscript was handled editorially. We look forward to future exciting submissions from your lab.

Sincerely,

Sarita Hebbar, PhD
Scientific Editor
Life Science Alliance
http://www.lsajournal.org
